# Perfusion fixation in brain banking: a systematic review

**DOI:** 10.1186/s40478-019-0799-y

**Published:** 2019-09-05

**Authors:** Whitney C. McFadden, Hadley Walsh, Felix Richter, Céline Soudant, Clare H. Bryce, Patrick R. Hof, Mary Fowkes, John F. Crary, Andrew T. McKenzie

**Affiliations:** 1 Department of Psychiatry, Icahn School of Medicine at Mount Sinai, One Gustave L. Levy Place, New York, NY 10029 USA; 2 Department of Pathology, Icahn School of Medicine at Mount Sinai, One Gustave L. Levy Place, New York, NY 10029 USA; 3 Graduate School of Biomedical Sciences, Icahn School of Medicine at Mount Sinai, One Gustave L. Levy Place, New York, NY 10029 USA; 4 Gustave L. and Janet W. Levy Library, Mount Sinai Health System, One Gustave L. Levy Place, New York, NY 10029 USA; 5 Nash Family Department of Neuroscience, Icahn School of Medicine at Mount Sinai, One Gustave L. Levy Place, New York, NY 10029 USA; 6 Friedman Brain Institute and Ronald M. Loeb Center for Alzheimer’s Disease, Icahn School of Medicine at Mount Sinai, One Gustave L. Levy Place, New York, NY 10029 USA; 7 Neuropathology Brain Bank and Research Core, Icahn School of Medicine at Mount Sinai, One Gustave L. Levy Place, New York, NY 10029 USA

**Keywords:** Brain banking, Perfusion fixation, Immersion fixation, Brain perfusion, Histology quality

## Abstract

**Background:**

Perfusing fixatives through the cerebrovascular system is the gold standard approach in animals to prepare brain tissue for spatial biomolecular profiling, circuit tracing, and ultrastructural studies such as connectomics. Translating these discoveries to humans requires examination of postmortem autopsy brain tissue. Yet banked brain tissue is routinely prepared using immersion fixation, which is a significant barrier to optimal preservation of tissue architecture. The challenges involved in adopting perfusion fixation in brain banks and the extent to which it improves histology quality are not well defined.

**Methodology:**

We searched four databases to identify studies that have performed perfusion fixation in human brain tissue and screened the references of the eligible studies to identify further studies. From the included studies, we extracted data about the methods that they used, as well as any data comparing perfusion fixation to immersion fixation. The protocol was preregistered at the Open Science Framework: https://osf.io/cv3ys/.

**Results:**

We screened 4489 abstracts, 214 full-text publications, and identified 35 studies that met our inclusion criteria, which collectively reported on the perfusion fixation of 558 human brains. We identified a wide variety of approaches to perfusion fixation, including perfusion fixation of the brain in situ and ex situ, perfusion fixation through different sets of blood vessels, and perfusion fixation with different washout solutions, fixatives, perfusion pressures, and postfixation tissue processing methods. Through a qualitative synthesis of data comparing the outcomes of perfusion and immersion fixation, we found moderate confidence evidence showing that perfusion fixation results in equal or greater subjective histology quality compared to immersion fixation of relatively large volumes of brain tissue, in an equal or shorter amount of time.

**Conclusions:**

This manuscript serves as a resource for investigators interested in building upon the methods and results of previous research in designing their own perfusion fixation studies in human brains or other large animal brains. We also suggest several future research directions, such as comparing the in situ and ex situ approaches to perfusion fixation, studying the efficacy of different washout solutions, and elucidating the types of brain donors in which perfusion fixation is likely to result in higher fixation quality than immersion fixation.

**Electronic supplementary material:**

The online version of this article (10.1186/s40478-019-0799-y) contains supplementary material, which is available to authorized users.

## Introduction

Much of our understanding of the pathophysiology of human diseases of the brain is derived from studies on postmortem human brain tissue [10, 15, 49]. The knowledge resulting from human postmortem brain research emphasizes the importance of collecting and banking the brains of human donors in as close to a life-like state as possible to allow for an accurate study of pathophysiologic processes. However, the methods used for banking human brain tissue are often not the methods that lead to the highest tissue quality. Therefore, there is a critical need to develop and optimize methods used to preserve human brain tissue. This will enable the full application of emerging three-dimensional brain tissue mapping methods that rely on the high-fidelity preservation of tissue architecture across large regions. These include spatial biomolecular profiling methods such as in situ transcriptomics [97], long-range circuit-tracing techniques using tissue clearing and immunostaining [72], and large-volume ultrastructural studies such as electron microscopy-based connectomics [101].

The two major methods for preparing human brain tissue for long-term storage are cryopreservation of small tissue blocks and chemical fixation of the tissue by crosslinking agents such as aldehydes [68, 80, 94]. Fresh-frozen tissue is essential for the study of brain biochemistry and has become especially important for brain banks over the past several decades, in part due to the flourishing of biochemical and molecular biological assays that require unfixed tissue [15, 49]. However, there are some studies, including ones that query cellular and tissue morphology, that are best performed on fixed tissue. There are two major methods for fixation: *immersion fixation*, which refers to placing the brain in a chemical bath that includes fixatives and waiting for the chemicals to diffuse into the brain tissue, and *perfusion fixation*, which refers to cannulating some part of the vasculature system and then driving fixative-containing fluid through the vessels, where it then travels out of circulation into the tissue. In human postmortem brain tissue, it has been estimated that it can take 20 to 46 days for a sufficient amount of formaldehyde to diffuse to the innermost parts of a brain hemisphere and begin fixation [21]. During this time, tissue in the inner regions of the brain will undergo microbial degradation, autolysis, breakdown of cellular membranes, and stochastic diffusion of molecules. As a result, immersion fixation causes gradients in fixation quality, whereby the surface regions where the fixation was initially applied has substantially better tissue preservation quality than deeper regions [5, 61]. However, in addition to simplicity, one upside of immersion fixation is that it does not rely on an intact neurovascular system, so the outermost surface millimeters of the brain tissue could undergo better fixation, especially if there are any clots occluding blood vessels.

Perfusion fixation of the brain has been performed in animal models for many decades as a way to preserve tissue integrity in a more robust and reliable manner [48]. Several investigators have compared perfusion fixation to immersion fixation for brain and vascular system fixation quality in animals, and these studies have generally found that tissues are substantially better preserved by perfusion fixation than immersion fixation beyond the first few millimeters, as measured by less displacement of neuropil, fewer vacuolar changes, and other metrics [17, 31, 50, 71]. While perfusion fixation is the gold standard for processing brain tissue prior to subsequent investigations in animals, it is not as commonly performed in contemporary human brain banking. Instead, one of the most common contemporary approaches to bank human brain tissue is to split the brain into two halves by making an incision at the midsagittal plane, and preserve one half via immersion fixation, and the other half via cryopreservation or freezing of small dissected portions of the brain [68, 80, 94]. Reasons that perfusion fixation is not as commonly used in banking human brain tissue include tissue and blood vessel damage that often occur prior to death and lack of access to equipment and relevant expertise by those procuring brain tissue. However, differences in fixation quality between immersion and perfusion fixation have been found to account for apparent differences in the nervous systems of humans and animals [54].

In this systematic review, we aimed to identify studies that have performed perfusion fixation for human brain tissue preservation and performed a qualitative synthesis of their methodologies. The major research questions we set out to answer were what methods have been used for human brain perfusion fixation and how does perfusion fixation compare to immersion fixation in terms of preservation outcomes. We attempted to contextualize the choices investigators made with reference to other literature, such as the literature on perfusion fixation of animal brains. The rationale of this review is to present a unified and accessible source of the experiences of researchers who have previously employed perfusion fixation in human brain tissue, for investigators who themselves may be interested in using the method. While systematic reviews have been published on the use of cadaver reperfusion for surgical training including neurosurgery training [8, 33, 100], to the best of our knowledge there has not been a review of methods for perfusion fixation in human brain tissue preservation. Our review revealed that while the method has been used since the 1960s, there is no clear trend of an increased use of this method in recent years. In terms of outcomes, the available evidence suggests that perfusion fixation probably leads to equivalent or improved subjective histology quality compared to immersion fixation of relatively large volumes of brain tissue, in a shorter amount of time.

## Methods

The systematic review was conducted following PRISMA (Preferred Reporting Items for Systematic Reviews and Meta-Analyses) guidelines. The protocol for the review and updated versions of it the can be found at Open Science Framework (https://osf.io/cv3ys/). The PRISMA checklist is also available (Additional file 1). During the review process, there were several changes made between the original protocol and the methods we employed. These are noted below in the section “Differences between the protocol and the review.”

### Search methods

We searched Embase Classic+Embase (1947 to February 2019), Medline All (1946 to February 2019), PubMed, and Scopus without language or date restriction (see Additional file 2 for detailed searches). The database search strategies include a combination of subject headings and keywords. To identify additional publications that are missed by these searches, we screened the references and citing articles (as identified by Scopus) of all included articles.

### Eligibility criteria

Any scholarly publication such as a journal article or textbook chapter that describes methods for perfusion fixation of the human brain was included. To be included, a study only needs to report on the perfusion fixation of human brain tissue and describe the methods for doing so; it does not need to be primarily about the process of perfusion fixation of the human brain. Fixation was defined as the use of a chemical substance or mixture of chemicals designed to preserve the tissue architecture and molecules in their lifelike state. Perfusion fixation was defined as using the vascular system in order to distribute fixatives throughout brain tissue. Studies on human brain tissue of any age were included. Studies were included if they perfuse the whole brain, only part of the brain such as a hemisphere, or a particular brain region. Studies that are performed by the same investigators and describe the same methods without substantive changes were considered together as one study, referred to by the study with the most detailed description of the methods. Studies written in any language were considered. If not written in English, studies were translated with the help of online tools such as Google Translate and Yandex Translate.

Although our focus is on the use of perfusion fixation for brain banking, our search strategy allowed us to identify articles that used perfusion fixation of postmortem human brain tissue for any type of research study, rather than only brain banking in particular. We used this approach to try to increase the pool of studies using perfusion fixation on human brain tissue from which we could learn and draw conclusions.

### Study selection

Using the online software Covidence, one reviewer (A.M.) screened the titles and abstracts identified by the searches and screened them in for further review on the basis of the eligibility criteria. Subsequently, two individuals (W.M. and A.M.) reviewed the full text of these articles, determined which articles met criteria for inclusion, and noted the exclusion reason(s) for the other articles. Disagreements were resolved by a consensus meeting.

### Data collection

For all included studies, at least two reviewers (H.W., F.R., C.B., and/or A.M.) extracted data variables about the methods and outcomes related to human brain perfusion fixation (see Additional file 3 for the questionnaire). In the case that there was disagreement between these reviewers that could not be addressed by further assessment of the manuscript by one of the reviewers (A.M.), then an additional reviewer (W.M.) was referred to in order to establish a decision. The data variables that were extracted are: number of perfusion- and immersion-fixed brains; exclusion criteria that would prevent the use of perfusion fixation for fixing brain tissue, for example long postmortem interval or vascular disease; tissue processing prior to vascular access; vessels accessed for perfusion; prefixative infusion; fixative mixture and buffer; time for perfusion; amount of fluid perfused; perfusion pressure; tissue processing before postfixation; postfixation procedure for perfusion fixed brains; tissue processing and storage procedure for perfusion fixed brains; metric(s) for fixation quality; downstream assays used or suggested; metric(s) for comparison to immersion fixation; and outcomes in comparison to immersion fixation. In the case that the variables were likely performed, known, or measured by the study authors but not reported, we attempted to contact the corresponding author(s) of the study via email and inquire about the variables.

### Study appraisal

Studies that present an explicit comparison between perfusion fixation and immersion fixation and/or between methods of perfusion fixation were assessed using the Joanna Briggs Institute (JBI) critical appraisal tool for quasi-experimental studies [91]. To harmonize the study appraisal tool with the downstream Cochrane tool for grading outcomes by the risk of bias of the studies included, we made one change to this checklist: we added an explicit question about the use of blinding by each study in the outcome assessment (Additional file 3). To maintain the same number of questions, we removed question #1 about clarity between “cause” and “effect,” which is not relevant to the experimental designs in the studies that we are assessing. For each of the studies, the number of “yes” answers out of the total number of questions was counted. “Not applicable” criteria were excluded, while criteria that were “unclear” were counted equivalently to a “no,” or not meeting the criteria. Studies were given an overall quality rating of “low” if 0–33% of the JBI questions were “yes,” “medium” if 34–66% of the criteria were “yes,” and “high” if 67% or more of the criteria were “yes.” Low quality studies were excluded from the outcomes grading step, as has been performed by a different systematic review using the JBI criteria [84]. The study quality metrics were assessed by at least two reviewers (H.W., F.R., C.B., and/or A.M.). In the case that there was a disagreement between these reviewers, an additional reviewer (W.M.) decided. The study quality metrics were taken into account when considering the strength of the evidence in the outcomes that they report.

### Qualitative data analysis

A qualitative survey of the different methods that have been reported for perfusion fixation in human brain banking was performed. Where possible, comparisons were made between the reported outcomes of immersion compared to perfusion fixation for brain banking. Because the studies were not expected to measure or report quantitative data on fixation quality, we performed a qualitative synthesis rather than a quantitative meta-analysis. Outcomes were evaluated using the GRADE (Grading of Recommendations, Assessment, Development, and Evaluations) method [75]. Each outcome between perfusion and immersion fixation was considered separately and had its own row in the summary of findings table. There were two outcomes assessed: (1) the subjective histology quality following either immersion or perfusion fixation and (2) the subjective histology quality following either immersion or perfusion fixation and after long-term storage in fixative. There are four possible levels for outcome quality in the GRADE method: high, moderate, low, and very low. In the GRADE method, all results derived from randomized trials start with a grade of high, while results derived from non-randomized studies start with a grade of low. Next, these grades were downgraded by one level for serious concerns or two levels for very serious concerns about risk of bias, inconsistency, indirectness, imprecision, and publication bias. They were upgraded by one level for large magnitudes of effect, for a dose-response relationship, or when the effects of all plausible confounds would go against the effect seen. The risk of bias for each study was assessed as a part of the JBI critical appraisal checklist. For example, confounding bias was assessed by the JBI checklist question about whether the participants in any comparisons were similar. Two reviewers (W.M. and A.M.) worked independently to evaluate the quality of evidence for each outcome and then came to a consensus decision.

### Differences between the protocol and the review

We note the following changes from the preregistered protocol. First, to grade the outcomes identified in the studies between perfusion and immersion fixation, we added these components to the questionnaire and methods. Critical appraisal of studies was only performed for studies that included a comparison between perfusion and immersion fixation, as the other studies were descriptive. In order to maintain the same appraisal criteria consistently across randomized and non-randomized experimental studies, all of the studies that compared perfusion fixation to immersion fixation or compared methods of perfusion fixation were critically appraised using the JBI checklist for quasi-experimental studies. Because it was not possible to adequately appraise studies that made only an *implicit* comparison between perfusion and immersion fixation, we changed the protocol so that only studies that made an *explicit* comparison were included in this section of the review.

One assumption made during the data extraction phase was that if the article described performing perfusion fixation on “brains” following autopsy, unless otherwise noted the study was assumed to have removed the brain from the skull prior to perfusion fixation and therefore was classified as “ex situ.” We also found that many of the studies listed brain donor exclusion criteria that were independent of the use of perfusion fixation but specific to their study needs, such as the absence of neurologic or psychiatric disease in a study of neurotypical brain tissue. Therefore, we attempted to identify brain donor exclusion criteria that were particular to the use of perfusion fixation.

After the data extraction process, we decided that the studies, methods, and outcomes for the comparisons between methods of perfusion fixation identified were too few and heterogeneous to provide any meaningful qualitative synthesis across studies. Therefore, we did not perform outcomes grading for comparisons between methods of perfusion fixation. We also did not identify any studies that compared how the perfusion fixation and immersion fixation approaches differed in fixation quality based on the brain tissue characteristics, so this was also not addressed. The outcomes selected for comparison between immersion and perfusion fixation were determined after the data extraction stage on the basis of the available data, and were not included in the original protocol. Finally, we also decided that studies that were deemed “low” quality based on our predetermined summary threshold of JBI quality metrics would not be included in the outcomes grading.

### Format of this review

The first part of this review section will list the methods for perfusion fixation used by the included studies, while the second part will summarize any outcomes of comparisons between perfusion and immersion fixation.

## Results and discussion

### Characteristics of included studies

**Fig. 1:**
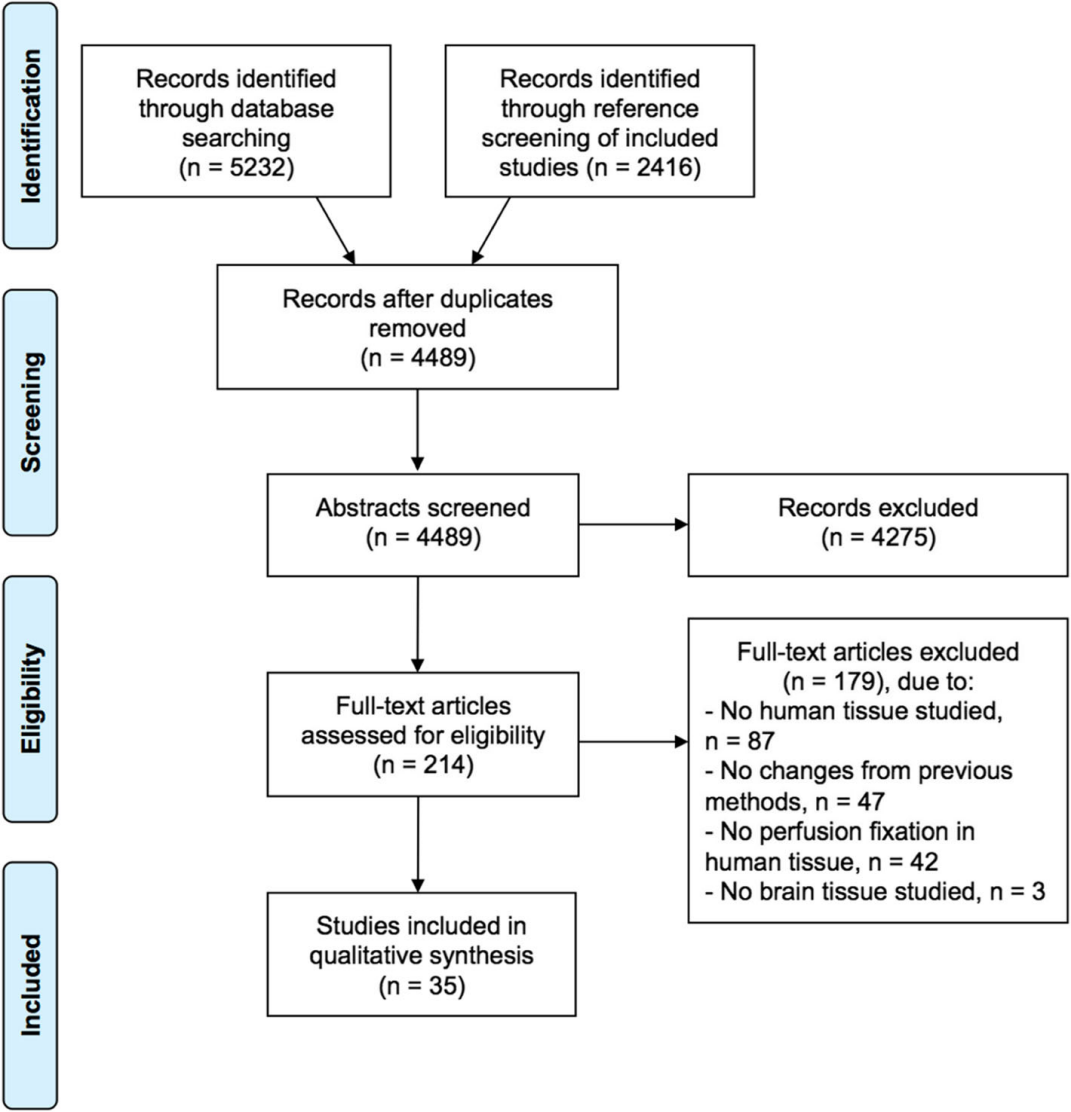
Study selection PRISMA flow diagram

We screened 4489 abstracts, 214 full-text publications, and identified 35 studies that met our inclusion criteria, which collectively reported on the perfusion fixation of 558 human brains (Fig. 1). Reasons for full-text exclusion decisions were that: no humans were studied (i.e. only animal models; 87 studies), no changes were made from previous methods (i.e.*,* another article that used the same methods was already was included; 47 studies), no perfusion fixation was performed in human tissue (e.g., perfusion fixation in animals and immersion fixation in humans; 42 studies), and brain tissue was not studied (e.g., only inner ear tissue studied; 3 studies).

**Fig. 2:**
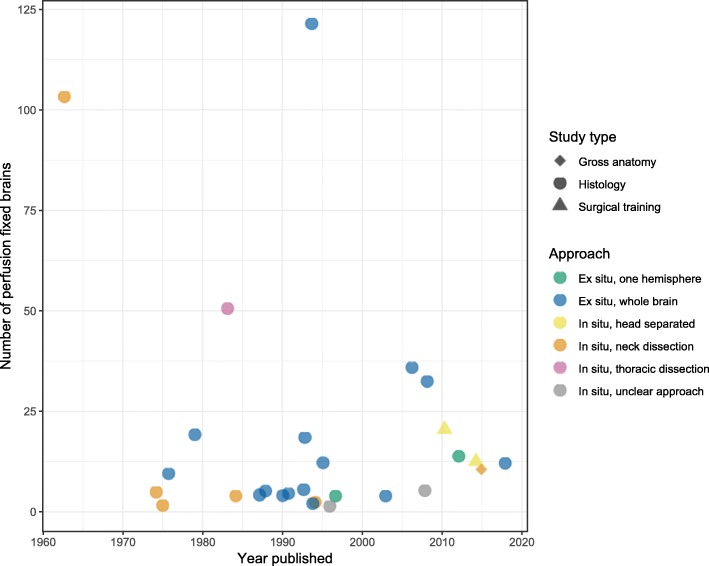
Characteristics of human brain perfusion fixation methods employed over time. Studies that had unclear approaches or did not report the number of perfusion-fixed brains are not drawn in the figure. This chart was prepared using R (v. 3.5.1) and the ggplot2 package

The studies were classified into three types: histology, e.g., for neuropathologic examination, forensic examination, or to study biomolecular and morphologic mechanisms of human brain function and disease; gross anatomical study, e.g. of white matter anatomy; or surgical training, e.g. for neurosurgery (Table 1). Of the articles focused on histology, there was an additional distinction between studies focused primarily on blood vessels (e.g., Lin et al. [57], Böhm [12], Masawa et al. (1993) [59], Shinkai et al. [81], Feekes et al. [28]) and studies focused primarily on brain parenchyma (e.g., Beach et al. [7], Halliday et al. [35], Welikovitch et al. [99], Donckaster et al. [24]). By plotting the methods used and the number of brains reported as perfused in each study, it is possible to examine qualitative trends over time, such as a relative decrease in the use of the in situ approach for histology studies (Fig. 2).

**Table 1:** General characteristics of included studies

Study	Country	Study type	Approach	Number of perfusion-fixed brains
Adickes 1996 [2]	United States	Histology	Ex situ, whole brain	NR
Adickes 1997 [1]	United States	Histology	Ex situ, one hemisphere	4
Alvernia 2010 [3]	France, United States	Surgical training	In situ, head separated	20
Beach 1987 [7]	Canada, Japan	Histology	Ex situ, whole brain	4
Benet 2014 [9]	United States	Surgical training	In situ, head separated	12
Böhm 1983 [12]	Germany	Histology	In situ, thoracic dissection	> = 50 (histology for 12)
Coveñas 2003 [20]	Spain	Histology	Ex situ, whole brain	4
de Oliveira 2012 [23]	Brazil	Histology	Ex situ, one hemisphere	14
Donckaster 1963 [24]	Chile, Uruguay	Histology	In situ, neck dissection	103
Feekes 2005 [28]	United States	Gross anatomy	Unclear	40
Grinberg 2008 [34]	Brazil	Histology	Ex situ, whole brain	32
Halliday 1988 [35]	Australia	Histology	Ex situ, whole brain	5
Huang 1993 [40]	Australia	Histology	Ex situ, whole brain	5
Insausti 1995 [42]	Spain	Histology	Ex situ, whole brain	12
Istomin 1994 [43]	Russia	Histology	Ex situ, whole brain; In situ, neck dissection	NR
Kalimo 1974 [45]	United States	Histology	In situ, neck dissection	5
Latini 2015 [53]	Sweden	Gross anatomy	In situ, neck dissection	10
Lin 2000 [57]	Japan	Histology	Unclear	NR
Lyck 2008 [58]	Denmark	Histology	In situ, unclear approach	5
Masawa 1993 [59]	Japan	Histology	Ex situ, whole brain	18
Masawa 1994 [60]	Japan	Histology	Ex situ, whole brain	121
McGeer 1988 [62]	Canada	Histology	Ex situ, whole brain	NR
McKenzie 1994 [64]	United States	Histology	In situ, neck dissection	2
Nakamura 1991 [69]	Japan	Histology	Ex situ, whole brain	4
Pakkenberg 1966 [70]	Denmark	Histology	In situ, unclear approach	1
Sharma 2006 [79]	United Kingdom	Histology	Ex situ, whole brain	36
Shinkai 1976 [81]	Japan	Histology	Ex situ, whole brain	9
Sutoo 1994 [85]	Japan	Histology	Ex situ, whole brain	2
Suzuki 1979 [86]	Japan	Histology	Ex situ, whole brain	19
Tanaka 1975 [87]	United States	Histology	In situ, neck dissection	1
Torack 1990 [90]	United States	Histology	Ex situ, whole brain	4
Turkoglu 2014 [92]	United States	Surgical training	In situ, head separated	NR
von Keyserlingk 1984 [93]	Germany	Histology	In situ, neck dissection	4
Waldvogel 2006 [96]	New Zealand	Histology	Ex situ, whole brain; Ex situ, single hemisphere	NR
Welikovitch 2018 [99]	Hungary, Canada	Histology	Ex situ, whole brain	12

For study type, we categorized each study into one of three types (histology, gross anatomy, or surgical training) based on our interpretation of the primary use of the tissue by each the investigators. Note that “histology” as the primary goal for a study is defined to include neuropathologic examination, forensic examination, or to study biomolecular and morphologic mechanisms of human brain function and disease. NR: Not reported

### Methods of perfusion fixation for brain banking

#### Approach to perfusion fixation

A major difference among studies that emerged was whether the investigators performed the perfusion fixation while the brain was still in the skull (i.e., in situ perfusion) or whether they removed the brain from the skull prior to performing the perfusion fixation (i.e., ex situ perfusion). There were two major subcategories for each approach. For the in situ approach, vessels were accessed either after making surgical incisions in the neck (or thorax) or after separating the head. For the ex situ approach, vessels were accessed either in the whole brain or in one isolated brain hemisphere. Two studies reported on multiple approaches. Istomin [43] reported methods for both ex situ whole brain and in situ neck dissection approaches, whereas Waldvogel et al. [96] reported methods for both ex situ whole brain and ex situ one-hemisphere approaches.

For the in situ approaches, one of the challenges described was difficulty perfusing the brain in the context of brain circulation deficits and/or brain trauma. Kalimo et al. [45] reported that in two of the five brains that they attempted to fix via perfusion, there was no fixation noted when the brain was removed; in both of these cases, there was evidence to suggest premortem deficits in circulation to the brain. Böhm [12], who performed the procedure on cadavers that had suffered injury to the head and brain, reported that the increased intracranial pressure resulting from brain death prevented cerebral perfusion throughout the internal carotid distribution. This was indicated by postmortem angiography that stopped at the intracranial internal carotid artery, which they called the “no-reflow phenomenon.” To mitigate this problem, Böhm [12] opened the skull and capped the upper half of the brain prior to perfusion fixation. This problem appears to be mitigated by using the ex situ approach. For example, Sharma et al. [79], who used the ex situ approach, reported perfusion fixation on brains donated from 5 individuals who had raised intracranial tension, or “pump brain,” prior to death. They found adequate or high-quality histology results when they did perfusion fixation on these brain samples.

Another challenge with the in situ approach is that it is more difficult to monitor perfusion fixation. Because the brain should harden during fixation, in an ex situ approach, it is possible to directly monitor fixation by applying pressure to the brain and noting resistance. In the in situ approach, the best monitoring method is likely fixation of the eyeball, which Donckaster et al. [24] and Latini et al. [53] both reported to be a suitable proxy for intracranial fixation. However, fixation of the eye may not always be completely reliable, due in part to the anastomosis between the external carotid and internal carotid through the ophthalmic artery. Kalimo et al. [45] reported that even after clamping the external carotid artery, partial fixation of tissues in the external carotid distribution would occur unless digital pressure was applied to the inner supraciliary skin and perfusion fixation was kept to a short period of time. Finally, a practical downside of the in situ approach is that it can interfere with funeral and embalming practices. For example, Istomin [43] noted that it was necessary to prepare the face of the cadaver prior to beginning the perfusion fixation, such as closing the eyes.

The in situ separated head approach was reported by 3 studies, all of which had the primary goal of surgical training. One consideration for the in situ separated head approach is the spinal level at which the head separation should be performed. Benet et al. [9] performed the separation at vertebral levels C5-C7 to allow for sufficient exposure of the cervical vessels, while retaining the cervical spinal cord.

For the ex situ approaches, one of the challenges described is the mechanical damage and deformation that occurs while the organ is removed from its regular location in the skull. In the animal literature, mechanical postmortem trauma has been found to result in histological artifacts such as dark neurons [44]. Investigators described several different approaches to minimize trauma. One approach is to suspend the brain in cloth; for example, Istomin [43] reported using a hammock of dense fabric for holding the brain in place. Another approach is to bathe the brain in liquid; for example, Beach et al. [7] placed the brain in phosphate-buffered saline. Beach et al. [7] reported that of these two methods, the liquid bath solution may lead to less mechanical damage. Another challenge with the ex situ approach is that the arteries can be easily damaged while handling the brain, which will make subsequent perfusion more challenging or impossible. Beach et al. [7] reported that when they removed the brain, they severed the carotid arteries so that there would still be long segments attached to the circle of Willis.

Regarding the ex situ one hemisphere approach, there are some special considerations. The process of cutting the brain introduces additional mechanical trauma that causes damage to the unfixed brain tissue and severs the arteries that supply the contralateral hemisphere, requiring additional artery ligations to prevent leakage of washout and fixative solution. Furthermore, the absence of collateral circulation from the contralateral circulation is likely to lead to worse overall fixation quality compared to the whole brain approach. In the process of cutting one hemisphere, it is also necessary to cut off the brainstem and cerebellum, with the result that these brain regions will not be perfusion-fixed because they are detached from the rest of the brain where the fixative is being perfused [95]. As a result of these problems, the ex situ one hemisphere approach is typically performed only in cases where the other hemisphere needs to remain unfixed, to preserve the tissue for biomolecular or biochemical studies.

Taken together, there were four major approaches to brain perfusion fixation reported, each of which have reported benefits and downsides, although there is very little data on comparisons among them.

#### Brain donor exclusion criteria for perfusion

Many of the studies listed criteria for the inclusion of brain tissue in their studies; however, it was almost always unclear whether these exclusion criteria were specific to the perfusion fixation preservation procedure rather than overall inclusion in the study. The one exception is Adickes et al. (1996) [2], in which cerebral vessel thrombosis or large intracerebral hemorrhages were both exclusion criteria specifically for perfusion fixation. In these cases, the investigators used immersion fixation. These exclusion criteria make biological sense, as these conditions are likely to interfere with flow through the cerebrovascular tree and therefore prevent adequate fixation.

While we did not identify any study that specifically noted that an extended postmortem interval (PMI) was an exclusion criterion for perfusion fixation, many of the studies reported the PMI range of the brain tissue used in their studies. The PMI range tolerated appeared to be associated with the goals of the investigators. On one extreme, Latini et al. [53], who studied gross anatomy of the white matter, reported that they tolerated a PMI of up to 7 days, which was the longest PMI range we identified among the included studies. At the other extreme, Kalimo et al. [45], who studied ultrastructure of brain parenchyma, used an “immediate autopsy” method such that their perfusion fixation procedure began within two minutes of death and the entire procedure was done within approximately 20 to 30 min after death. Another study of ultrastructure, by Suzuki et al. [86], also required brain donors with a relatively short PMI of less than 5 h. They noted that autopsy cases after 5 h demonstrated worse preservation of the cytoplasm or cellular organelles, including vacuolar and liquefaction changes, which they attributed to autolysis. Somewhere in the middle of these extremes fell the majority of the light microscopy-based immunohistochemistry studies. For example, Beach et al. [7] reported that they achieved “satisfactory” staining with PMIs of up to 18 h, although they noted that their immunohistochemistry results were best with brain tissue less than 12 h postmortem. As another immunohistochemistry example, Halliday et al. [35] performed perfusion fixation on brains with PMIs of up to 35 h.

In summary, cerebral vessel thrombosis or large intracerebral hemorrhages were the only exclusion criteria specific to perfusion fixation. Several studies also suggested that a short PMI was preferred, with the PMI range tolerated depending on the type of the downstream study.

#### Vessels accessed for perfusion

Among the studies that we evaluated, there were many different choices in the vessels that they accessed for subsequent perfusion steps, which depended on the overall approach that they employed (Table 2). A key trade-off is ease of vascular access and technical perfusion quality versus the degree of dependence on intact collateral circulation for reaching more distant brain regions.

**Table 2:** Vascular access strategies reported by the included studies

Study	Approach	Vessels Accessed	Cannula	Vessels Occluded
Adickes 1996 [2]	Ex situ, both hemispheres	Unilateral vertebral artery, bilateral carotid arteries	18G cannula	Contralateral vertebral artery
Adickes 1997 [1]	Ex situ, one hemisphere	Internal carotid artery; if the PCoA was too small or not present, second cannula placed in the posterior cerebral artery	18G plastic cannula	Basilar and contralateral cerebral arteries
Alvernia 2010 [3]	In situ, head separated	Common carotid arteries, vertebral arteries, internal jugular veins	One-way urinary catheter (largest possible)	NR
Beach 1987 [7]	Ex situ, whole brain	Bilateral internal carotid arteries, bilateral vertebral arteries or basilar artery	Plastic IV cannula	NR
Benet 2014 [9]	In situ, head separated	Common carotid arteries, vertebral arteries, jugular veins	NR	NR
Böhm 1983 [12]	In situ, thoracic dissection	Aortic arch	Wide balloon catheter	NR
Coveñas 2003 [20]	Ex situ, whole brain	Carotid and vertebral arteries	NR	NR
de Oliveira 2012 [23]	Ex situ, one hemisphere	Internal carotid artery, posterior communicating artery*	20G peripheral catheter*	Basilar artery* and contralateral hemisphere arteries
Donckaster 1963 [24]	In situ, neck dissection	Bilateral carotids, with or without vertebral arteries	Irrigation cannula	External carotids
Feekes 2005 [28]	Unclear	Carotid artery	NR	NR
Grinberg 2008 [34]	Ex situ, whole brain	Bilateral internal carotid arteries and vertebral arteries*	Olive C cannula*	NR
Halliday 1988 [35]	Ex situ, whole brain	Carotid and vertebral arteries	NR	NR
Huang 1993 [40]	Ex situ, whole brain	Bilateral internal carotid arteries and vertebral arteries	NR	NR
Insausti 1995 [42]	Ex situ, whole brain	Both internal carotids, if both PCoAs were sufficient diameter; One carotid and the basilar artery otherwise	NR	Non-cannulated arteries were ligated
Istomin 1994 [43]	Ex situ, whole brain	Internal carotid arteries and basilar arteries	NR	NR
Istomin 1994 [43]	In situ, neck dissection	Bilateral carotid arteries	NR	NR
Kalimo 1974 [45]	In situ, neck dissection	Initial segment of the right internal carotid artery	Glass cannula	Right external carotid, both left carotid arteries, and vertebral arteries
Latini 2015 [53]	In situ, neck dissection	Left or right common carotid artery	NR	NR
Lyck 2008 [58]	In situ, unclear approach	Internal carotid artery	NR	NR
Masawa 1993 [59]	Ex situ, whole brain	Bilateral internal carotid arteries	NR	NR
Masawa 1994 [60]	Ex situ, whole brain	Bilateral carotid arteries	NR	NR
McKenzie 1994 [64]	In situ, neck dissection	Bilateral common carotid arteries	Polyethylene cannula (1/4″ outside diameter)	Vertebral arteries and internal jugular veins (intermittently clamped)
Nakamura 1991 [69]	Ex situ, whole brain	Bilateral internal carotid and vertebral arteries	NR	NR
Pakkenberg 1966 [70]	In situ, unclear approach	Unilateral carotid artery	NR	NR
Sharma 2006 [79]	Ex situ, whole brain	Blood vessels at the base of the brain and floor of the third ventricle (non-vessel)	NR	NR
Shinkai 1976 [81]	Ex situ, whole brain	Bilateral internal carotid and vertebral arteries	NR	NR
Sutoo 1994 [85]	Ex situ, whole brain	Bilateral internal carotid arteries and basilar artery	NR	NR
Suzuki 1979 [86]	Ex situ, whole brain	Bilateral middle cerebral arteries	NR	NR
Tanaka 1975 [87]	In situ, neck dissection	Left internal carotid artery	NR	NR
Torack 1990 [90]	Ex situ, whole brain	Bilateral internal carotid arteries and the basilar artery	NR	After initial perfusion fixation, clamped vessels to isolate the hippocampus
Turkoglu 2014 [92]	In situ, head separated	Bilateral internal carotid arteries	One-way number 10 Foley urinary catheters	External carotid arteries
von Keyserlingk 1984 [93]	In situ, neck dissection	Internal carotid artery, vertebral artery	NR	NR
Waldvogel 2006 [96]	Ex situ, whole brain	Basilar and internal carotid arteries	21G winged infusion needles	Leaking vessels occluded
Waldvogel 2006 [96]	Ex situ, one hemisphere	Internal carotid, vertebral, and anterior cerebral arteries	21G winged infusion needles	Leaking vessels occluded
Welikovitch 2018 [99]	Ex situ, whole brain	Internal carotid and vertebral arteries	Serum 1 needle*	NR

The overall approach to perfusion fixation, blood vessels cannulated, cannula type used, and any vessels reported as clamped or otherwise occluded by the included studies. If an included study did not describe the vessels that were accessed, it is not listed here. Asterisks indicate personal communications. NR: Not reported, PCoA: Posterior communicating artery

All of the included studies attempted to perfuse the anterior circulation of the brain via the carotid artery distribution in some form; either via the common carotid artery or arteries, internal carotid artery or arteries, or the aortic arch. Waldvogel et al. [96] also reported cannulation of the anterior cerebral artery in their ex situ one hemisphere approach. If only one side of the two carotid arteries is cannulated for perfusion, then interhemispheric collateral circulation will likely provide some fixative to the other hemisphere via the anterior communicating artery [55]. However, the perfusion quality in that hemisphere will be limited, especially if the anterior communicating artery is absent or hypoplastic [78]. In the in situ approach, if the internal carotid was cannulated, several of the investigators (Table 2) also clamped the external carotid to prevent shunting of perfusate to the often lower-pressure external carotid circulatory distribution, as opposed to the brain.

Slightly more than half (20/32 or 62.5%) of the included studies reported consistently cannulating vessels in the posterior circulation in some form; either the vertebral artery or arteries, basilar artery, posterior cerebral artery, or the aortic arch. The remainder of the studies either did not focus on brain regions supplied by the posterior circulation or relied on collateral circulation from the anterior to the posterior circulatory system. Collateral circulation via the posterior communication arteries is not intact in approximately one-fifth of people [102], although some degree of leptomeningeal collateral circulation may still be present [73]. Notably, the ability to visualize the posterior communicating arteries directly is an advantage of the ex situ approach, as the likely amount of collateral circulation through the circle of Willis can be visually assessed and the vessels to perfuse chosen accordingly (performed by Insausti et al. [42] and Adickes et al. (1997) [1]).

For obvious reasons, it is technically easier to cannulate fewer arteries, and this also decreases the time interval for tissue degradation prior to the initiation of washout and fixation. Cannulating more arteries also potentially affects perfusion quality within each one of the arteries when using a perfusion setup with a tube splitter to distribute the perfusate, as was used in Beach et al. [7]. This is because perfusion flow will distribute to the lowest pressure arteries, and cannulating a low-pressure artery that distributes fixative to a less important region of the brain may lead to worse quality fixation in a more important region of the brain. Finally, one of the advantages of the ex situ approach is that it is easier to access more blood vessels on the ventral surface of the brain without requiring more extensive neck dissection to access the vertebral artery. Relatively more of the studies using the ex situ than the in situ neck dissection approach reported consistently cannulating at least one artery in the posterior circulatory system (Table 2).

One study, Sharma et al. [79], reported perfusion fixation via the lateral ventricles using the ex situ approach, in addition to the blood vessels. This method likely allowed for improved fixation of periventricular brain structures such as the hypothalamus. The lateral ventricular perfusion method was also used with good reported results by Toga et al., who used an in situ approach and was not identified by our formal search methods [89]. This study found that their intraventricular delivery system led to better and more uniform fixation preservation quality than perfusion of fixatives through the carotid and vertebral arteries. They speculated that this was due to erratic blood clot formation during the postmortem interval.

Torack et al. [90] reported a unique procedure in an attempt to isolate the hippocampus as a target for perfusion fixation. They first perfused through the internal carotid arteries and the basilar artery. Next, they clamped the middle cerebral artery distal to the anterior choroidal artery and the posterior cerebral artery distal to the posterior choroidal arteries. Following these occlusions, the perfusion fixation should have been more targeted to the hippocampus.

The main goal of vascular access points in perfusion fixation is to perfuse a large portion of the brain with little damage to the tissue. The studies that were able to successfully cannulate the anterior circulation as well as the posterior circulation would likely perfuse the largest amount of brain tissue. We are unable to determine if the quality of the tissue isolated from brains with different perfusion access protocols is significantly different.

#### Washout solution used

Slightly more than half (20/35 or 57%) of the included studies reported using a washout solution prior to perfusion fixation (Table 3). This step aims to remove clots, blood cells, and other intravascular debris to improve flow of fixative, although it comes at the cost of increased procedural complexity and a longer delay prior to fixation. Adickes et al. (1997) [1] did not use a “pre-perfusion” or washout step with saline because it would make the procedure more burdensome on staff. Donckaster et al. [24] only used their washout solution in cases with a PMI of more than 12 h prior to the initiation of the procedure, with the goal of preventing the fixation of blood clots. Of the studies that employed a washout step, saline or phosphate-buffered saline were the most common base washout solutions used, while two of the studies used mannitol, and one study used Ringer solution.

**Table 3:** Washout solutions used by the included studies

Study	Base solution	Additives	Drive method	Time	Amount	Rate	Pressure	Stopping criterion
Alvernia 2010 [3]	Warm tap water	NR	Syringe (60 ml)	NR	2–4 l	NR	NR	Until water flow was clear (clot/debris removal)
Beach 1987 [7]	Ice cold PBS	NR	Pump	10–20 min	1 l	50–100 ml/min	NR	NR
Benet 2014 [9]	Isotonic saline	NR	NR	NR	NR	NR	“Low pressure”	Until contralateral outflow was clear
Böhm 1983 [12]	Ringer solution in 0.2 M phosphate buffer (pH 7.5)	Rheomacrodex (Dextran 40)	Gravity	5–10 min	5 l	500–1000 ml/min	NR	Until blood and blood clots were washed away
Coveñas 2003 [20]	0.15 M PBS (pH 7.2)	NR	Pump	NR	1 l	NR	NR	NR
de Oliveira 2012 [23]	Mannitol	Warm heparin	Gravity	NR	250 ml	NR	NR	NR
Donckaster 1963 [24]	Physiological saline	NR	NR	NR	NR	NR	NR	NR
Grinberg 2008 [34]	NaCl 0.9%	NR	Gravity*	NR	NR	NR	147.4 mmHg (height of 2 m*)	NR
Grinberg 2008 [34]	20% Mannitol	Heparin	Gravity*	NR	250 ml	NR	147.4 mmHg	NR
Halliday 1988 [35]	0.1 M Sodium phoshate (pH 7.4)	1% sodium nitrite	Pump	NR	5 l	NR	“Normal mean arterial pressure”	NR
Huang 1993 [40]	PBS	NR	NR	33 mins	4 l	120 ml/min	NR	NR
Insausti 1995 [42]	Saline at 4 °C	Heparin, 10,000 units	NR	20 mins	2 l	100 ml/min	NR	NR
Istomin 1994 [43]	Saline	NR	Gravity or Syringe	NR	NR	NR	150 mmHg	Clear fluid flow from the veins
Kalimo 1974 [45]	NaCl 0.9%	NR	Gravity	<= 5 mins	NR	NR	NR	NR
Lin 2000 [57]	0.01 M PBS (pH 7.4)	NR	NR	NR	NR	NR	NR	NR
Nakamura 1991 [69]	0.01 M sodium-PBS (pH 7.4)	NR	Pump	NR	1 l	NR	NR	NR
Sutoo 1994 [85]	Ice cold PBS (pH 7.4)	NR	NR	NR	2 l	NR	NR	NR
Torack 1990 [90]	PBS	NR	NR	NR	180 ml (60 ml in each vessel)	NR	NR	NR
Turkoglu 2014 [92]	Saline	NR	Gravity	NR	3 l	NR	110 mmHg (height of 1.5 m)	Until no visible blood or clots drained from the IJVs
Waldvogel 2006 [96]	PBS (pH 7.4)	1% sodium nitrite	Pump	15 mins	0.5 l	~ 33 ml/min	NR	15 min or until the brain is cleared of blood
Welikovitch 2018 [99]	Physiological saline	0.33% heparin	Gravity	30 mins	1.5 l	50 ml/min	NR	NR

If the study is not listed here, then it did not report the use of a washout solution. If gravity was used to drive perfusate, we used the formula P = ρgh, where P = hydrostatic pressure, ρ = density of substance (assumed equal to water), g = gravitational acceleration, and h = height, to calculate the pressure. Asterisks indicate personal communications. NR: Not reported, PBS: Phosphate-buffered saline, IJV: Internal jugular vein

Published perfusion fixation methods for laboratory animals often start while the animal is anesthetized [30]. This protocol prevents substantial premortem and postmortem clot formation [36], which means that the major purpose of the washout solution is to remove blood cells from the vessels. On the other hand, in postmortem human brain perfusion fixation, there is frequently an abundance of blood clots that limit perfusion quality [22]. This means that in addition to washing out the cells, the washout step is often used by investigators to also decrease the clot burden by driving them out with pressure. Böhm [12] noted that the washout step removed most clots that had formed postmortem, while clots that were formed premortem could only be washed out if a higher perfusion pressure was employed. Notably, the goal of Böhm [12] was to *preserve* premortem clots for forensic purposes, whereas studies using perfusion fixation to study brain parenchyma typically aimed to remove clots in order to improve perfusate flow and resulting fixation quality.

In addition to mechanically removing blood clots via perfusion pressure, another approach is to degrade or inhibit clots enzymatically. Four of the studies added the anticoagulant heparin to their washout solution, which may help to limit the spread of blood clots (Table 3). One of the studies, Böhm [12], reported the occasional use of dextran 40, which also has antithrombotic properties [74].

Two of the studies, Halliday et al. [35] and Waldvogel et al. [96], reported the addition of sodium nitrite to the washout solution. Sodium nitrite may help to dilate blood vessels and has been found to improve perfusion fixation quality in animals [71].

The volume of the washout solution varied considerably, from as little as 180 ml to as much as 5 l. Several of the studies also reported performing the washout step until the venous outflow was clear of blood, clots, or debris.

One potential problem with the use of a washout solution in brain perfusion fixation is that it may induce brain edema. In animal studies it has been shown that perfusing too much saline into the brain (e.g., one liter) can cause edema [11]. The edema induced may be related to the osmotic concentration of the washout solution. Consistent with this, Benet et al. [9] found that washing out with an isotonic saline solution rather than tap water led to decreased tissue edema. Grinberg et al. [34] compared a hyperosmolar solution of 20% mannitol with a solution of 0.9% NaCl, finding that 20% mannitol led to substantially less brain swelling. Böhm [12] also used a hyperosmolar washout solution (680 mOsm) composed of Ringer solution in 0.2 M phosphate buffer.

Overall, the majority of articles included a washout step, most commonly using 1–5 l of saline as the base washout solution. The additives used and the precise procedure reported differed widely, and there were few comparisons between methods.

#### Fixative solution used

Consistent with its widespread use throughout pathology and histology, formaldehyde was a component of the fixative used in almost all studies. The only exceptions were one condition in Grinberg et al. [34] that employed 70% ethanol only (which did not lead to successful fixation) and 3 studies that used glutaraldehyde only (Table 4). Some studies used paraformaldehyde, which is a polymerized storage form of formaldehyde, while others used formalin, which is a form of formaldehyde that includes methanol to inhibit polymerization. 10% formalin is composed of 3.7% formaldehyde with around 1% or less of methanol [88]. Paraformaldehyde typically requires depolymerization via heating and/or sodium hydroxide prior to use, thus adding another setup step that adds complexity and will potentially prolong the interval prior to the initiation of the procedure [47]. The addition of methanol in formalin keeps the formaldehyde depolymerized and avoids its precipitation.

**Table 4:** Fixative solutions reported by the included studies

Study	Fixative solution	Buffer	Drive	Time	Amount	Flow rate	Pressure
Adickes 1996 [2]	10% buffered formalin	NR	Gravity	15–20 min	2 l	100–133 ml/min	75.6 mmHg (height of 1 m)
Adickes 1997 [1]	10% buffered formalin	Phosphate	Gravity	15–20 min	2 l	100–133 ml/min	75.6 mmHg (height of 1 m)
Alvernia 2010 [3]	Formaldehyde 37% and ethyl alcohol 10%	NR	Syringe (60 ml)	NR	NR	NR	NR
Beach 1987 [7]	4% paraformaldehyde (ice cold)	0.1 M phosphate buffer (pH 7.4)	Pump	40–80 min	4 l	50–100 ml/min	NR
Benet 2014 [9]	10% formaldehyde	NR	NR	NR	0.7 l	NR	NR
Benet 2014 [9]	Custom solution: ethanol 62.4%, glycerol 17%, phenol 10.2%, formaldehyde 2.3%, and water 8.1%	NR	NR	NR	0.7 l	NR	NR
Böhm 1983 [12]	2% glutaraldehyde	0.2 M phosphate buffer	Gravity	5–10 min	5–10 l	~ 1000 ml/min	25.7–47.8 mmHg
Coveñas 2003 [20]	4% paraformaldehyde	0.15 M PBS (pH 7.2)	NR	NR	3 l	NR	“Normal mean arterial pressure”
de Oliveira 2012 [23]	20% formalin	NR	Gravity	NR	5 Ll	NR	NR
Donckaster 1963 [24]	Cajal fixative: formalin and ammonium bromide	NR	NR	NR	900 ml (300 ml in children < 12 years old)	NR	< 200 mmHg
Feekes 2005 [28]	10% formalin	NR	NR	NR	NR	NR	NR
Feekes 2005 [28]	2.5% formaldehyde, 6% isopropyl alcohol, 1% glycerin	NR	NR	NR	NR	NR	NR
Grinberg 2008 [34]	10% formalin	None	Gravity	NR	5 l	NR	147.4 mmHg (height of 2 m*)
Grinberg 2008 [34]	20% formalin	None	Gravity	NR	5 l	NR	147.4 mmHg
Grinberg 2008 [34]	70% ethanol	None	Gravity	NR	5 l	NR	147.4 mmHg
Grinberg 2008 [34]	Acetic acid-alcohol-formalin	None	Gravity	NR	5 l	NR	147.4 mmHg
Halliday 1988 [35]	4% formaldehyde, 2% picric acid; followed by 10% sucrose in fixative	0.1 M sodium phosphate	Pump	NR	10 l fixative only; 4 l 10% sucrose in fixative	NR	“Normal mean arterial pressure”
Huang 1993 [40]	4% paraformaldehyde	0.1 M phosphate buffer	NR	83 mins	10 l	120 ml/min	NR
Insausti 1995 [42]	4% paraformaldehyde (4 °C) or 4% paraformaldehyde, 0.02% picric acid (4 °C)	NR	NR	120 mins	4 l or 8 l	33 or 67 ml/min	NR
Istomin 1994 [43]	10–12% formalin	Neutral buffered	Syringe or Gravity	NR	NR	NR	150 mmHg
Kalimo 1974 [45]	1.0% paraformaldehyde, 2.0% glutaraldehyde (37 °C)	0.1 M cacodylate (pH 7.4)	Gravity	NR	1.5 l (adult), 0.7 l (newborn)	NR	132 mmHg
Latini 2015 [53]	12% formalin	NR	Infusion device (compressed air mechanism)*	15–20 min	2 l	100–133 ml/min	1500 mmHg (200 kPa)
Lin 2000 [57]	4% paraformaldehyde, 0.2% picric acid, and 0.1% glutaraldehyde	0.1 M phosphate buffer (pH 7.4)	NR	NR	NR	NR	NR
Lyck 2008 [58]	4% formalin	75 mM phosphate buffer (pH 7.0)	NR	NR	NR	NR	NR
Masawa 1993 [59]	4% formalin, 1% glutaraldehyde	0.1 M phosphate buffer (pH 7.4)	NR	NR	400 ml	NR	100 mmHg
Masawa 1994 [60]	10% buffered formalin	NR	NR	NR	NR	NR	100 mmHg
McGeer 1988 [62]	4% paraformaldehyde, 0.1% glutaraldehyde	0.1% phosphate buffer (pH 7.4)	NR	NR	NR	NR	NR
McKenzie 1994 [64]	10% formalin	Neutral buffered	Gravity	60 mins	12–14 l	200–233 ml/min	75.6 mmHg (height of 1 m)
Nakamura 1991 [69]	4% paraformaldehyde, 0.1% glutaraldehyde (ice cold)	0.1 M phosphate buffer (pH 7.4)	Pump	15 mins	1 l	70–80 ml/min	NR
Pakkenberg 1966 [70]	Alcohol 80% 9 parts, formalin 4% 1 part	NR	NR	NR	NR	NR	NR
Sharma 2006 [79]	20% formalin	Neutral buffered	NR	NR	NR	NR	NR
Shinkai 1976 [81]	2.5% glutaraldehyde containing 0.2 M sucrose	0.1 M phosphate buffer (pH 7.4)	NR	NR	NR	NR	NR
Sutoo 1994 [85]	4% paraformaldehyde, 0.2% glutaraldehyde	PBS	NR	90 mins	6 l	67 ml/min	NR
Suzuki 1979 [86]	2.5% glutaraldehyde	Phosphate buffer (pH 7.4)	NR	5–10 min	NR	NR	NR
Tanaka 1975 [87]	2% glutaraldehyde, 1% paraformaldehyde (pH 7.2)	0.1 M sodium cacodylate	NR	NR	0.7 l	NR	NR
Torack 1990 [90]	4% paraformaldehyde (4 °C)	0.1 M phosphate buffer (pH 7.4)	NR	30 mins	1.68 l (560 ml in each artery)	50 ml/min	“40 lbs. of pressure”
Turkoglu 2014 [92]	10% formaldehyde	NR	Gravity	60 mins	NR	NR	110.4 mmHg (height of 1.5 m)
von Keyserlingk 1984 [93]	1% paraformaldehyde, 1% glutaraldehyde, 1.65% potassium dichromate	0.1 M cacodylate buffer (pH 7.4)	NR	NR	5 l	NR	NR
Waldvogel 2006 [96]	15% formalin	0.1 M phosphate buffer (pH 7.4)	Pump	30–45 min	2 l	~ 33 ml/min	NR
Welikovitch 2018 [99]	4% paraformaldehyde, 0.05% glutaraldehyde, and 0.2% picric acid	0.1 M phosphate buffer	Gravity*	90–120 min	4–5 l	33–56 ml/min	NR

This table lists the fixatives solutions and their buffers, amounts, times for perfusion, flow rates, methods for driving perfusate, and/or perfusion pressures that are reported by the included studies. If gravity was used to drive perfusate, we used the formula P = ρgh, where P = hydrostatic pressure, ρ = density of substance (assumed equal to water), g = gravitational acceleration, and h = height, to calculate the pressure. Asterisks indicate personal communications. NR: Not reported, PBS: Phosphate-buffered saline

Twelve of the studies employed glutaraldehyde in the perfusion solution, at various concentrations ranging from 0.05% in Welikovitch et al. [99] to 2.5% in Shinkai et al. [81] and Suzuki et al. [86]. In general, adding glutaraldehyde to the fixative solution allows for improved tissue morphology preservation for electron microscopy [67], at the cost of decreased immunogenicity of antigens for immunohistochemistry [47]. However, at lower concentrations of glutaraldehyde, such as the 0.05% used in Welikovitch et al. [99], its effects on antigenicity are likely to not be as pronounced, and it likely acts primarily to slightly improve tissue morphology.

In addition to formaldehyde and glutaraldehyde, some investigators have used other fixatives. Picric acid, also known as 2,4,6-trinitrophenol, was used by Halliday et al. [35] (2%), Insausti et al. [42] (0.02%), Lin et al. [57] (0.2%), and Welikovitch et al. [99] (0.2%). Picric acid has been found to improve preservation of immunogenicity compared to aldehyde fixation alone [82], although safety concerns make this fixative less desirable due to its explosive properties.

Pakkenberg et al. [70] used a solution made up of 9 parts 80% alcohol and 1 part 4% formalin, which fixed the tissue to a quality sufficient for counting the number of nucleoli in the cortex, but also led to 20% volume shrinkage. This is consistent with the dehydrating effect of alcohol fixatives [39]. Other studies that used alcohol in their fixative solutions included Feekes et al. [28], Grinberg et al. [34], and Benet et al. [9].

Two of the studies used sucrose as a component of their perfused fixative solution, Shinkai et al. [81] and Halliday et al. [35]. The addition of sucrose might help to optimize the osmotic concentration of the perfusate [13, 98] and/or to act as a cryoprotectant to prevent tissue morphology changes due to ice damage during sectioning with a freezing microtome.

Donckaster et al. [24] perfused Cajal fixative, which consists of formalin and ammonium bromide. The addition of ammonium bromide is thought to facilitate silver staining of neural cells [52]. von Keyserlingk et al. [93] perfused 1% paraformaldehyde, 1% glutaraldehyde, and 1.65% potassium dichromate. The addition of potassium dichromate has been found to aid in the fixation of lipids [38], which is consistent with the focus of von Keyserlingk et al. [93] on myelin ultrastructure.

Benet et al. [9] used a custom fixative composed of ethanol 62.4%, glycerol 17%, phenol 10.2%, formaldehyde 2.3%, and water 8.1%, which they compared to a fixative with 10% formaldehyde for use in surgical simulation. They concluded that the custom fixative was superior for surgical simulation, in part because it caused less hardening and therefore allowed for more realistic tissue retraction.

Grinberg et al. [34] compared four different fixatives in their study. They found that perfusion of 20% formalin and acetic acid-alcohol-formaldehyde both led to efficient fixation of deep brain structures, while 10% formalin did not, and 70% ethanol did not harden at all. However, they found that the acetic acid-alcohol-formaldehyde fixative led to dissolution of myelin, while 20% formalin did not.

The fixative vehicle or buffer can also have important effects on tissue preservation [16]. The most common buffer in the studies we identified was phosphate buffer, which was reported in 19 of the studies. Phosphate buffer can be titrated to maintain an approximately neutral pH, at which point the fixative solution can also be called “neutral-buffered.” One of the most important aspects of the buffer is the molarity, which is thought to be the major driver of the osmotic concentration of the fixative solution [14]. Although there is some controversy on this point, aldehyde fixatives themselves are generally not considered major drivers of the osmotic concentration, as they easily cross semipermeable cell membranes, and therefore do not exert a sustained osmotic force [37]. As a result, the osmotic concentration of the fixative vehicle is called the effective osmotic concentration. Hypertonic fixative solutions can cause grossly shrunken brain tissue and cell shrinkage, whereas hypotonic solutions can cause edema and resistance to flow in the perfusion procedure [77].

It would be convenient to be able to identify the optimal vehicle osmotic concentration that would minimize osmotic tissue changes. However, Böhm [12] pointed out that the redistribution of fluids and ions during hypoxia makes it difficult to identify this optimal osmotic concentration in the postmortem state, which is consistent with more recent evidence [46, 51]. To study this empirically, Böhm [12] used fixative solutions with multiple different osmolarities, finding that a mildly hypertonic solution with a total osmotic concentration of 500 mOsm and an effective osmotic concentration of 300 mOsm led to the best fixation quality in their study.

Several of the included studies manipulated the temperature of their fixative solution prior to perfusion. Beach et al. [7] cooled their fixative solution to be “ice-cold,” while Torack et al. [90] and Insausti et al. [42] cooled their fixative solution to 4 °C. Lower temperatures can help to inhibit metabolism and thereby mitigate tissue degradation, although it has also been reported to cause vasoconstriction [29]. One study, Kalimo et al. [45], perfused their fixative at the elevated temperature of 37 °C, which has been suggested to facilitate vasodilation and improve perfusion flow [29].

Taken together, 1–10 l of phosphate-buffered formaldehyde was the most common fixative solution perfused. The most important determinants of the fixative are the assay of interest and the tissue or cell type of interest (e.g. neurons or myelin). The choice of fixative buffer is an important way to balance tissue shrinkage and swelling while the fixative is being perfused and can affect fixation quality.

#### Driving perfusate and perfusion pressure

The three major methods for driving the flow of solution during perfusion are syringes, gravity, and perfusion pumps. All three methods were reported by the included studies: 2 studies reported using a syringe, 8 studies reported using gravity, and 4 studies reported using a pump (Table 4). The majority of studies did not report their drive method. Upsides of a syringe are that it is easier to inject a specific amount of fluid in each vessel, while it is more difficult to control flow rate and pressure.

From the perspective of a perfusion circuit, the included studies were open-circuit in that they did not describe using a method for re-introducing the outflow of the perfusate back into the vessels. In the in situ approaches, the perfusate typically drained from the internal jugular veins after flowing through the carotid and/or vertebral circulatory systems. In the ex situ approaches, the perfusate would be expected to drain from the cerebral veins and/or ruptured vessels below the isolated brain, for example into a container.

A major trade-off in setting the perfusion pressure is that too high of a perfusion pressure may lead to a higher risk of vessel rupture [76], while too low of perfusion pressure may lead to incomplete perfusion, decreased clot removal, and decreased tissue penetration of the fixative [17]. In laboratory animals, investigators often suggest that perfusion pressure should be maintained at roughly the same pressure that it was during life, which is called physiologic pressure [25, 30]. Consistent with this, Halliday et al. [35] and Coveñas et al. [20] reported that their perfusion pressures were “normal mean arterial pressure.” Böhm [12] kept their perfusion pressure lower, in the range of 25.7 mmHg (35 cm H_2_O) to 47.8 mmHg (65 cm H_2_O), because they were concerned that the endothelium is less stable postmortem than it is while the person is alive. However, Latini et al. [53] used the supraphysiologic pressure of 1500 mmHg (200 kPa) to study white matter anatomy, and they were able to preserve and dissect white matter blood vessels of submillimeter size.

Techniques using syringes, gravity, and perfusion pumps have all been employed to drive perfusion flow at a variety of different pressures. However, there were no studies that made comparisons between these alternative methods or identified an optimal perfusion pressure range for a particular application.

#### Postfixation procedures

In the context of perfusion fixation, “postfixation” refers to immersion fixation of the tissue sample for some amount of time following the initial perfusion, either in the original fixative or in a new fixative solution. The procedure for postfixation depends on whether the perfusion fixation was perfused in situ or ex situ (Table 5). If in situ, then the brain was often left in the skull for some amount of time to allow for fixative diffusion prior to removal. This time period ranged from 1 h in McKenzie et al. [64], 1 to 2 h in Kalimo et al. [45], and 2 h in von Keyserlingk et al. [93], to 48 h in Latini et al. [53].

**Table 5:** Postfixation procedures reported by the included studies

Study	Pre-processing	Fixative	Buffer	Temp	Length of postfixation
Adickes 1996 [2]	NR	10% buffered formalin	Phosphate	NR	1 day (if postfixed)^a^
Adickes 1997 [1]	Cut into 1–1.5 cm-thick sections	10% buffered formalin	Phosphate	NR	5–6 h
von Keyserlingk 1984 [93]	Brain left in skull for 2 h, then removed and dissected	1% osmium tetroxide	0.1 M sodium cacodylate	NR	2 h
Istomin 1994 [43]	NR	10–12% formalin	Neutral-buffered	NR	NR
Beach 1987 [7]	NR	4% paraformaldehyde	0.1 M phosphate buffer (pH 7.4)	4 °C	NR
Benet 2014 [9]	NR	1:10 dilution of 10% formaldehyde	NR	5 °C	> = 2 days
Benet 2014 [9]	NR	1:10 dilution of 10% custom solution (ethanol 62.4%, glycerol 17%, phenol 10.2%, formaldehyde 2.3%, and water 8.1%)	NR	5 °C	> = 2 days
Böhm 1983 [12]	Cut into 1 cm-thick coronal sections	Paraformaldehyde or formalin	0.1 M phosphate buffer	NR	NR
Coveñas 2003 [20]	NR	4% paraformaldehyde	0.15 M PBS (pH 7.2)	4 °C	30 days
de Oliveira 2012 [23]	NR	20% formalin	NR	NR	3 weeks
Donckaster 1963 [24]	Brain removed	Cajal fixative: formalin and ammonium bromide	NR	NR	4 days
Grinberg 2008 [34]	NR	Same fixative as was used for fixation	NR	NR	NR
Huang 1993 [40]	Dissection of brainstem	NR	NR	NR	<= 24 h
Insausti 1995 [42]	Dissected into slabs approximately 1 cm thick	4% paraformaldehyde	NR	NR	48–72 h
Kalimo 1974 [45] (electron microscopy)	Brain left in the skull for 1 to 2 h after perfusion fixation, then removed, then samples dissected for EM	1.0% paraformaldehyde, 2.0% glutaraldehyde	0.1 M cacodylate (pH 7.4)	NR	Overnight
Kalimo 1974 [45] (histology)	Same as above	10% formaldehyde	NR	NR	10 days
Latini 2015 [53]	Brain extracted from the skull 48 h after perfusion	10% formalin	NR	NR	24 h
Lin 2000 [57]	NR	4% paraformaldehyde	0.1 M phosphate buffer (pH 7.4)	4 °C	Overnight
Lyck 2008 [58]	Brain removed from skull	4% paraformaldehyde	0.15 M Sørensens phosphate buffer (pH 7.4)	4 °C	2 weeks
Masawa 1993 [59]	NR	4% formalin	0.1 M phosphate buffer	NR	> = 3 days
Masawa 1993 [59] (electron microscopy)	From postfixed tissue, tissue blocks were cut and buffer washed	1% osmium tetroxide solution	NR	4 °C	90 min
McGeer 1988 [62]	NR	4% paraformaldehyde	NR	NR	2–3 days or until the pink color of unfixed erythrocytes was gone
McKenzie 1994 [64]	Waited 1 h after perfusion fixation, then the skull was opened, and the brain was removed	Formalin	Neutral-buffered	4 °C	NR
Pakkenberg 1966 [70]	Brain removed from skull	Alcohol 80% 9 parts, formalin 4% 1 part	NR	NR	3 weeks
Sharma 2006 [79]	Brain suspended in a bucket	20% formalin	Neutral-buffered	NR	1–4 days
Shinkai 1976 [81]	Cut into 2 mm-thick tissue blocks	2.5% glutaraldehyde containing 0.2 M sucrose	NR	NR	4–8 h
Sutoo 1994 [85]	Brain halved sagittally and sliced into 10 mm coronal blocks	4% paraformaldehyde	PBS	4 °C	2 days
Suzuki 1979 [86]	Dissected bifurcations of the first temporal branches of the middle cerebral arteries	2.5% glutaraldehyde	NR	NR	4 h
Tanaka 1975 [87] (electron microscopy)	Samples taken from various regions of the brain	1.0% osmium tetroxide	NR	NR	NR
Tanaka 1975 [87] (histology)	Rest of the brain	8.0% formaldehyde	NR	NR	NR
Torack 1990 [90]	Hippocampus and entorhinal cortex was isolated and sectioned into 0.5 cm thick slices	4% paraformaldehyde +/− 1% Bouin’s solution (picric acid, acetic acid, and formaldehyde)	NR	NR	48 h
Turkoglu 2014 [92]	Brain removed from skull	10% formaldehyde	NR	NR	2 weeks
Waldvogel 2006 [96]	NR	15% formalin	0.1 M phosphate buffer (pH 7.4)	NR	6–12 h
Welikovitch 2018 [99]	Dissected out the medial temporal lobe	4% paraformaldehyde and 0.2% picric acid	0.1 M phosphate buffer	NR	Overnight

^a^: Note that in Adickes et al. (1996), the brain is either cut immediately or postfixed in formalin for one day. NR: Not reported, PBS: Phosphate-buffered saline

Many of the studies reported cutting the brain prior to additional postfixation; for example, in Nakamura et al. [69], the tissue was cut into 1–2 cm-thick coronal blocks. Perfusion-fixed tissue is harder and therefore easier to cut than fresh tissue. Cutting the tissue makes the subsequent immersion fixation process faster because there is a shorter distance for the fixatives to diffuse, with the obvious issue of damaging tissue at the cut interfaces.

There was a wide range of time frames used for postfixation, ranging from 4 h in Suzuki et al. [86] and 5–6 h in Adickes et al. (1997) [1] to 3 weeks in de Oliveira et al. [23] and Pakkenberg et al. [70] and 30 days in Coveñas et al. [20]. How long investigators chose to postfix for may depend in part on their perception of the quality of their perfusion fixation. One major advantage of postfixation is that it will allow for fixation even in regions of the brain where perfusion has been minimal or absent, for example as a result of persistent blood clots.

A key trade-off in the length of postfixation is that longer amounts of time will lead to better fixative penetration of deeper regions of the brain or tissue block, while it may also lead to over-fixation and decreased antigenicity in the outer regions of the brain (i.e., the cerebral cortex) or tissue block. As a result, a significant disadvantage of a long period of postfixation is that immunohistochemical staining and quantification will result in variable gradients across the tissue section. However, these gradients can be minimized by pre-processing steps that cut the tissue into smaller sections prior to postfixation. For example, Shinkai et al. [81] cut the tissue into 2 mm sections and Torack et al. [90] cut the tissue into 5 mm sections prior to postfixation.

The majority of the studies used the same fixative for perfusion fixation and postfixation. One exception is glutaraldehyde fixation studies, which typically omitted it from the postfixative, likely in order to mitigate further antigen masking. Another exception is three studies that prepared tissue samples for electron microscopy, Tanaka et al. [87], Masawa et al. (1993) [59], and von Keyserlingk et al. [93], which postfixed in osmium tetroxide, a fixative that stabilizes the ultrastructure of lipids and cell membranes [26].

In summary, postfixation is used commonly and it allows investigators to compensate for the possibility of poor perfusion quality. There was a wide range of postfixation procedures reported, ranging in time from a few hours to several weeks.

#### Long-term storage methods

Storing the brain in formaldehyde for the long-term prior to use is an economical and convenient way to prevent microbial and autolytic degradation. It is especially convenient for gross tissue preservation for surgical training, as was performed in Alvernia et al. [3] and Benet et al. [9]. However, for histology purposes, storage in formaldehyde has been found to lead to a decrease in antigenicity over time. Lyck et al. [58], who used this storage method, performed a quantitative study of several antigens over time, showing that antibody staining quality decreased for certain sensitive antigens, such as NeuN and CNPase, when stored in fixative over time. Similarly, McGeer et al. [62] noted that brains fixed in formalin for a long period of time had negative staining results for the protein that they were studying, HLA-DR.

An alternative method for long-term storage for subsequent histology is to store tissues at sub-zero temperatures. However, this method requires the distribution of cryoprotectant throughout the tissue to prevent ice damage. Four studies reported using this method for long-term storage (Table 6). Notably, the glycerol-dimethylsulfoxide cryoprotectant method used by Insausti et al. [42] has been found in non-human primate brain tissue to cause less tissue shrinkage than the sucrose-based methods [27].

**Table 6:** Strategies for long-term storage of perfusion-fixed brain tissue

Study	Overall method	Study type	Tissue	Preservative agent(s)	Temperature	Storage duration
Alvernia 2010 [3]	Immersion in fixative	Surgical training	Separated head	10% Formalin and 10% ethyl alcohol	4 °C	Up to 4 years
Benet 2014 [9]	Immersion in fixative	Surgical training	Separated head	10% formaldehyde or 10% custom solution (ethanol 62.4%, glycerol 17%, phenol 10.2%, formaldehyde 2.3%, and water 8.1%)	5 °C	Up to a year
Insausti 1995 [42]	Cryoprotection and freezing	Histology	1 cm-thick coronal tissue blocks	Solutions of 10 and 20% glycerol in 0.1 M phosphate buffer and 2% dimethylsulfoxide	−80 °C	NR
Lyck 2008 [58]	Immersion in fixative	Histology	Whole brain	0.1% paraformaldehyde in 0.15 M Sørensens phosphate buffer (pH 7.4)	4 °C	Up to 4 years
Sutoo 1994 [85]	Cryoprotection and freezing	Histology	1 cm-thick coronal tissue blocks	Buffered 5% sucrose	−80 °C	NR
Waldgovel 2006 [96]	Cryoprotection and freezing	Histology	Tissue blocks (many 1 cm-thick)	20–30% sucrose in 0.1 M phosphate buffer with 0.1% sodium azide	−80 °C	NR
Welikovitch 2018 [99]	Cryoprotection and freezing	Histology	Brain sections	1.1 M sucrose, 37.5% ethylene glycol in PBS	−20 °C	NR

If a study did not report the use of a long-term storage method, then it is not included in this table. NR: Not reported, PBS: Phosphate-buffered saline

To summarize, fixed brain tissue can be stored in fixative at refrigerator temperatures near 4 °C, but this will likely lead to a decrease in antigenicity over time. An alternative approach, which may allow for the preservation of antigenicity for longer, is to add cryoprotectant to the fixed brain tissue and store it at a freezer temperature such as − 80 °C.

### Comparisons of perfusion fixation with immersion fixation

#### Study selection

For 6 studies, at least two reviewers agreed that the study made an explicit comparison between immersion and perfusion fixation. For one of these studies, Adickes et al. (1996) [2], this outcome was graded as “low quality” on the basis of our risk of bias appraisal tool, as all of the applicable components for risk of bias were either graded as “unclear” or “no.” Therefore this study was removed, leaving 5 studies (Table 7).

**Table 7:** Description of studies with an explicit comparison between perfusion and immersion fixation

Study	Design	Number of brains fixed		Time for procedure		Outcome	Result
		Perfusion	Immersion	Perfusion	Immersion		
Adickes 1997 [1]	Crossover, within-brain	4	4	5–6 h	2 weeks	Subjective histology quality	Equal or superior tissue preservation with perfusion fixation compared with immersion fixation
Beach 1987 [7]	Experimental, non-randomized	2	2	1–8 days	1–8 days	Subjective histology quality	More even distribution of staining in perfusion-fixed samples, while immersion fixed samples had a dense band of staining at the edges of the fixed tissue and pale regions in the interior
Grinberg 2008 [34]	Experimental, non-randomized	32	4	Not reported	> 3 months	Subjective histology quality	More uniform penetration of fixative agent into all regions of the brain in perfusion-fixed samples, including deep regions such as the thalamus and basal ganglia
Lyck 2008 [58]	Experimental, non-randomized	32	5	1 day - 4 years	1 day - 10 years	Long-term immunostaining	Better preservation of sensitive antigens (e.g., NeuN and CNPase) in perfusion-fixed specimens
Sharma 2006 [79]	Experimental, randomized selection of brain tissue	36	36	1–4 days	3–4 weeks	Subjective histology quality	No significant difference in staining quality between perfusion and immersion fixation

Note that “histology quality” refers to visual microscopy results, including slides that have been stained with dyes as well as with antibody staining. Regarding the time for the procedure, note that in Beach et al. [7], the tissue was sliced into 1 cm-thick blocks prior to the postfixation or initial immersion fixation. In Lyck et al. [58], the time reported includes the time for long-term storage in fixative beyond the initial fixation procedure

#### Methodologies and results of included studies

Adickes et al. (1997) [1] performed a type of crossover study, using immersion and perfusion fixation on each hemisphere of the same autopsied brains. Sharma et al. [79] randomly selected slides from brain tissue that had previously been fixed with either immersion or perfusion fixation and then did prospective analysis of their histology quality via blinded reviewers. These are both considered optimal methodologies that were considered equivalent to a randomized study. The other 3 studies did not describe their methods for allocating donor brains to different interventions and were classified as non-randomized experimental studies.

The outcome described by the 4 of the studies, Adickes et al. (1997) [1], Beach et al. [7], Grinberg et al. [34], and Sharma et al. [79] was the immediate subjective histology quality following a perfusion fixation procedure compared to an immersion fixation procedure. Because the Sharma et al. [79] and Adickes et al. (1997) [1] studies had more optimal study methodologies, their results were weighted higher in the grading process in evaluating this outcome. The outcome of Lyck et al. [58] addressed antigen staining results for brain samples stored in fixative long-term that were initially perfusion fixed compared to those initially immersion fixed.

For the outcome of immediate subjective histology quality, Adickes et al. (1997) [1] found equal or superior histology quality in perfusion-fixed tissue, Sharma et al. [79] found no significant difference, while Grinberg et al. [34] and Beach et al. [7] found improved histology quality in perfusion-fixed tissue, especially in deep brain regions. Notably, the immersion fixation protocol was performed on the whole brain in Grinberg et al. [34] and Sharma et al. [79], one hemisphere in Adickes et al. (1997) [1], and 1 cm-thick blocks in Beach et al. [7]. Sharma et al. [79] used a scoring system in which staining from conventional fixed brains was taken as the gold standard, which we believe refers to immersion fixed brain tissue. For Adickes et al. (1997) [1] and Sharma et al. [79], the perfusion fixation protocol was also completed much faster than the immersion fixation protocol. Overall, these results can be summarized as showing that there is equal or superior subjective histology quality in perfusion-fixed samples in an equal or shorter amount of time, when compared to immersion fixation of relatively large volumes of brain tissue, such as the whole brain, one brain hemisphere, or 1 cm-thick tissue sections. When we mention time in this context, we are referring to the total time for the brain tissue to bathe in fixative during immersion fixation or postfixation before it is ready for downstream studies. In contrast, the time required for a trained worker to perform the procedure will almost certainly be longer for the perfusion-based methods.

For the outcome of immunostaining in samples stored in fixative long-term, Lyck et al. [58], found that there was better preservation of sensitive antigens (e.g., NeuN and CNPase) in perfusion-fixed specimens compared to immersion-fixed samples.

#### Risk of bias assessment

**Fig. 3:**
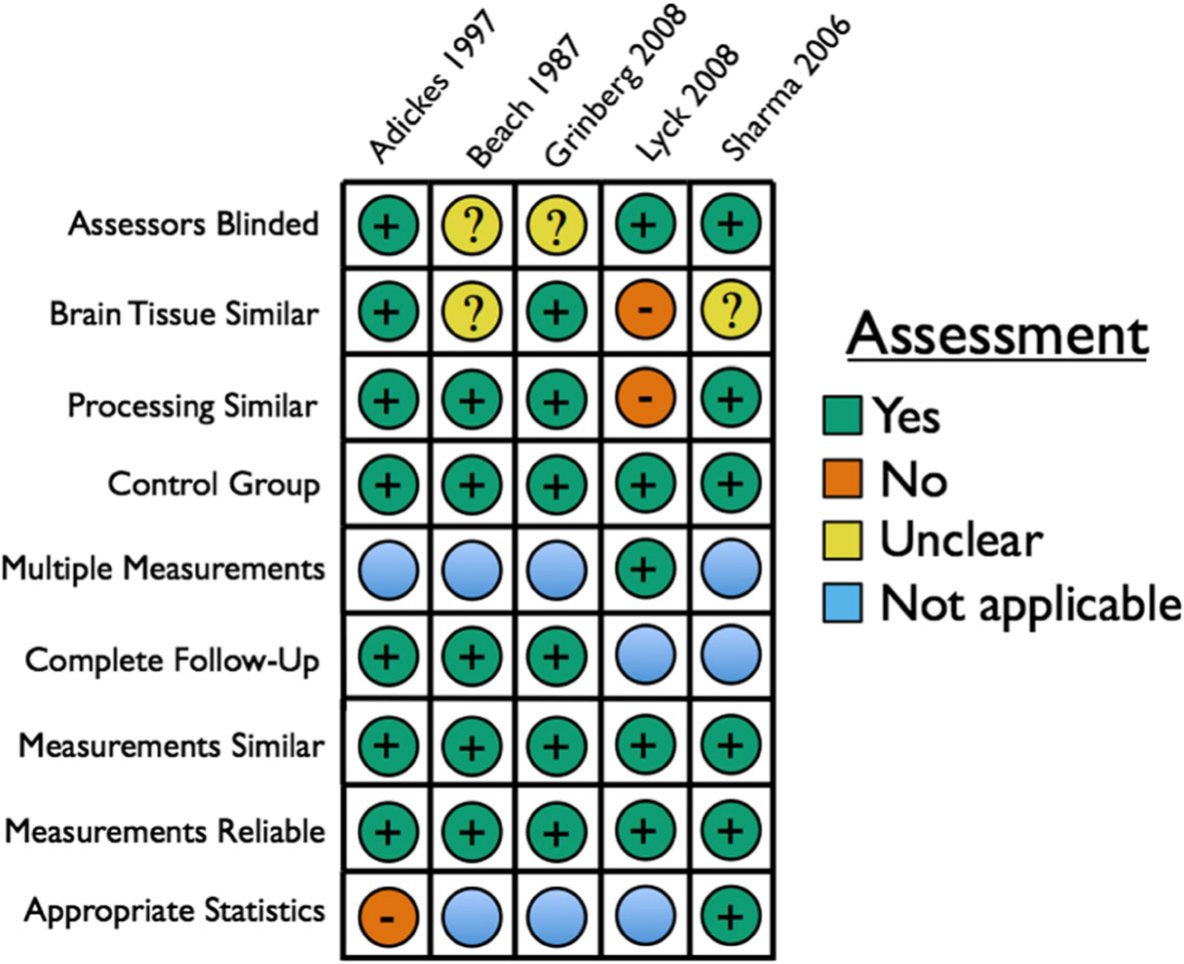
Risk of bias assessment for the studies comparing perfusion to immersion fixation. We used a modified version of the Joanna Briggs Institute (JBI) questionnaire for non-randomized experimental studies

During the data extraction process, at least two independent reviewers appraised the included studies on the JBI quality metrics (Fig. 3). Three of the studies reported blinding of the histology quality assessors, while this was not mentioned for the other two studies. For the confounding question, Beach et al. [7] and Sharma et al. [79] did not report on enough demographic and clinical data that would allow us to determine whether the brain tissue was of substantially similar quality prior to the procedures. Lyck et al. [58] had their brain tissue from different brain banks and the PMIs also differed substantially between the perfusion and immersion fixation groups. Lyck et al. [58] also used different processing on the immersion- and perfusion-fixed tissue, such as storing the brains at room temperature and the perfusion-fixed brains at 4 °C, which introduced another source of confounding bias. Overall, using our predefined summary of the risk of bias questionnaire, all five of the studies were assessed as being “high quality.”

#### Evidence grading

For the outcome of subjective histology quality immediately following the procedures, we assigned an evidence grade of moderate quality (Table 8). Because of the study methodologies of Adickes et al. (1997) [1] and Sharma et al. [79], the evidence grade started at high quality. The reason for downgrading this to moderate was imprecision, which came in two forms. First, the sample sizes were relatively small, especially in Adickes et al. (1997) [1] and Beach et al. [7], which used only 4 brains each. Second, while experts such as neuropathologists assessed the histology quality grades, these scores are semiquantitative. Future work that identifies and quantifies particular features present in each of the histology images would allow for more precise testing of differences in fixation quality between the different methods.

**Table 8:** Summary of findings for the two outcomes from comparing perfusion fixation and immersion fixation identified in our review

Outcome	Number of studies	Number of brain samples	Overall effect	Quality of the evidence (GRADE)
Subjective histology quality	4 (1 randomized, 1 crossover)	116	Equal or superior histology quality in perfusion-fixed tissues when compared to immersion fixation of relatively large volumes of brain tissue	⨁⨁⨁⨀ MODERATE
Long-term immunostaining quality	1	37	Slower long-term degradation of antigen staining quality for sensitive antigens in perfusion-fixed tissue	⨁⨀⨀⨀ VERY LOW

In the GRADE quality assessment system, there are four levels of quality: high, moderate, low, and very low

Aside from the time required to perform perfusion fixation and possible osmotic or hydrostatic effects on tissue resulting from the perfusion process, the main difference between perfusion fixation and immersion fixation is in the time needed for postfixation. Therefore, perfusion fixation can be thought of as a shift along the fixation time-fixation quality curve, such that there is an improvement of histology quality following a given duration of immersion fixation or postfixation. This strength of this shift will vary based on the quality of the perfusion fixation. In the extreme case of ideal perfusion fixation, postfixation may not be necessary, but human brain tissue quality is often compromised by the time it reaches a brain bank, for example by a long PMI, which will typically prevent ideal perfusion fixation.

For the outcome of long-term immunostaining quality in initially perfusion-fixed or immersion-fixed brain tissue stored in fixative, the one study identified, Lyck et al. [58], found that there was less long-term degradation of antigen staining quality for sensitive antigens in perfusion-fixed tissue. We assigned this outcome an evidence grade of very low quality based on the available evidence (Table 8) because of serious concerns of imprecision from low sample size (*n* = 5 perfusion-fixed brains), as well as serious concerns of confounding from heterogeneous tissue processing.

#### Informal comparisons reported between immersion and perfusion fixation

The studies that did not make a formal comparison between immersion and perfusion fixation or were assessed as having a low-quality study design regarding this comparison, often remarked on differences between these two fixation methods. Adickes et al. (1996) [2] noted that histology quality was “excellent” in perfusion-fixed tissue and was better than tissue fixed *en bloc* via immersion. Kalimo et al. [45] reported that perfusion fixation led to higher quality cellular and tissue-level preservation than immersion fixation, especially in deep brain regions. Insausti et al. [42] reported that perfusion fixation led to faster and more homogenous fixation. Von Keyserlingk et al. [93] noted that perfusion fixation had a more “satisfying” ultrastructural preservation of myelin in preliminary studies compared to immersing the brain in 5% formaldehyde. Torack et al. [90] reported that it was possible to identify dopaminergic fibers in the hippocampus via their perfusion fixation method, similar to observations in rodent studies, but not previously identified in immersion-fixed tissue. These informal comparisons support the use of perfusion fixation for the most complete fixation of brain tissue, although the purpose and aims of the study should be evaluated individually while determining the fixation method.

### Comparison to other reviews

To the best of our knowledge, there has not been a previous systematic review focused on the topic of perfusion fixation in human brain tissue. There has been a previous systematic review of perfusion techniques for surgical training [8]; however, it did not focus on perfusion fixation and histology quality in particular. One narrative review of one institution’s experience with brain banking notes that perfusion fixation is the optimal method, but that it is time-consuming and that immersion fixation of 1 cm-thick blocks at 4 °C is a reasonable alternative [6]. A response to Adickes et al. (1997) [1] by Miller [66] pointed out problems with perfusion fixation including artifactual perivascular pallor on myelin staining, difficulty in perfusion in the presence of ischemic infarcts or hemorrhagic tissue, and potentially increased exposure to formalin vapors. Another narrative review that was not specific to brain tissue noted that the literature contains conflicting evidence about whether perfusion fixation yields improved morphologic quality when compared to immersion fixation [4].

One book chapter by Connolly et al. [19] describes experience that perfusion speeds the fixation process and can improve immunohistochemical staining. However, corroborating one of the critiques of Miller [66], they note that perfusion fixation can occasionally cause irregular white matter pallor on hematoxylin and eosin stain that is likely artifactual. Connolly et al. [19] also note that in the presence of vascular diseases such as atherosclerosis, inadvertent damage to the circle of Willis during brain removal, and/or in cases of suspected cerebral emboli or thrombi, perfusion fixation can be difficult or even impossible. Another book chapter by Giannini et al. [32] also discusses experience in perfusion fixation of human brain tissue. They note that perfusion improves fixation of deeper regions of the brain. Giannini et al. [32] also point out several potential problems with perfusion fixation, including inducing gross asymmetry due to uneven perfusion of too much fixative in cases of an infarct, hemorrhage, or metastasis, artifactual dilatation of small blood vessels, and microscopic evidence of perivascular tissue rarefaction.

### Limitations of this review

One limitation of this review is the potential for publication bias. Immersion fixation is the standard method for brain banking via fixation, which means there is less incentive for authors to publish articles showing that immersion fixation is superior to perfusion fixation. Perfusion fixation critiques were found in less formal media, such as textbook chapters or short review articles. While this is less likely to affect our outcome grades because we did not find evidence that any studies explicitly comparing perfusion and immersion fixation were not published, it is important when considering the positive tone that many of the authors have towards the use of their own methods.

Furthermore, we certainly underestimated the total number of studies of perfusion fixation for human brain tissue preservation. One major reason for this is because we searched titles and abstracts rather than full-texts. Through ad hoc reviews of the literature, we were able to identify multiple studies [41, 83, 89] that used perfusion fixation in human brain banking but did not describe it in the title or abstract. However, as additional studies would have had diminishing returns in the variance of their methods employed, our review is still likely to have good coverage of the types of methods that have been used.

Finally, multiple changes were made to the protocol after the initiation of the review process. As a part of this, we did not pre-specify the outcomes to grade for methods comparison, which means that our choice of outcomes to grade could be more influenced by our biases based on their direction of effect. In part this is due to the relative paucity of previous literature on human brain perfusion fixation. This meant that we started the review process with relatively little knowledge of what types of data we would encounter and what types of outcomes would be reported and possible to grade.

### Recommendations for further research

One of the areas in which more research could make a major contribution is comparison between perfusion fixation methods. For example, on the broadest level, it would be interesting to see whether there is higher histology quality in the in situ approaches, which minimize mechanical trauma and blood vessel damage, or the ex situ approaches, which allow for direct monitoring of the washout and fixation procedures and may be more robust to raised intracranial pressure. Comparisons between different washout solutions would also be valuable, as the accumulation of perimortem clots that frequently occlude perfusion is relatively unique to human brain banking [36]. For example, it might be useful to test whether washout with reagents used in perfusing transplant organs, such as fibrinolytic agents [56], might allow for improvements in perfusion fixation. Additionally, if it is possible to develop perfusion fixation methods that are less expensive, time-consuming, and/or technically challenging compared to immersion fixation, this may help more brain banks to adopt the method.

The comparison studies that we identified in this review all performed immersion fixation on relatively large volumes of brain tissue, with a minimum size of 1 cm-thick tissue blocks. Further research could evaluate the extent to which slicing the tissue into smaller volumes, such as a thickness of 5 mm or less, prior to immersion fixation might allow for improved histology quality compared to perfusion fixation. In addition, it may be possible to accelerate immersion fixation through other approaches, such as using high-frequency ultrasound to enhance fixative delivery [18].

Another area for improvement of perfusion fixation as a brain banking procedure is in improving methods for the long-term storage of fixed brain tissue. For example, a study could compare different cryoprotectants for preserving human tissue morphology and antigenicity in slices at low temperature, as has been performed in non-human primate brain tissue [27]. Another useful storage method could involve perfusing cryoprotectants after the fixative perfusion, to facilitate storage of intact brain tissue at low temperatures [63, 65]. This would potentially allow for more detailed studies of cross-region neuronal connectivity or ex situ neuroimaging with fewer batch effects resulting from long-term storage in formalin. Regarding ex situ neuroimaging, another question for future research is whether images taken from brains fixed with perfusion would allow for a higher correlation with pre-mortem images than brains fixed with immersion.

Some of the included studies and reviews of this topic suggested that perfusion fixation should not be performed on brain tissue with certain characteristics, such as cerebrovascular disease or long PMI. However, these claims are often not supported by direct data, and other studies cast doubt upon some of them; for example, Waldvogel et al. [96] noted the relative importance of periagonal factors as opposed to the PMI in determining immunohistochemical staining quality. Further research that characterized the types of brain tissue in which it is beneficial to use perfusion fixation as opposed to immersion fixation would be valuable.

## Conclusions

Our systematic review of perfusion fixation for human brain banking discovered that a wide variety of methods have been used. The earliest studies reporting human brain perfusion fixation primarily used the in situ approach, but since the mid-1970s, the ex situ approach has become more common. In order to allow half of the brain to be frozen for biomolecular or biochemical studies, a more recent innovation over the past two decades has been to perform perfusion fixation on only one isolated hemisphere of the brain. For neuropathologists and investigators in brain banks, we identified moderate quality evidence that perfusion fixation leads to equal or higher subjective histology quality in relatively large volumes of brain tissue, while taking equivalent or less time than immersion fixation. However, perfusion fixation has been reported to have some downsides, including potential for tissue edema or uneven fixation in the presence of cerebrovascular disease. Furthermore, there are substantial logistical, technical, and financial challenges involved in perfusion fixation that are not required by the relatively simple method of immersion fixation. Improvements in the methods for perfusion fixation of human brain tissue would allow for novel investigations of human brain disease pathophysiology, such as high-resolution ex vivo neuroimaging, spatial biomolecular profiling, circuit tracing, and connectome studies.

For investigators running brain banks interested in using perfusion fixation, we can offer a few suggestions. First, it is important to acknowledge that many of the recent advances in our understanding of the pathophysiology of brain disease have come from studying frozen unfixed tissue [15, 49]. As a result, frozen unfixed tissue will remain a critical component of most brain banking protocols. For investigators who desire to bank a substantial amount of fresh unfixed tissue, the ex situ one hemisphere approach, despite its limitations, is the only feasible option. On the other hand, for investigators who are exclusively interested in studying fixed brain tissue, either the in situ or ex situ approaches may be worthwhile to consider. If minimal technical challenge is desired, then an ex situ approach employing gravity to drive the perfusion of standard formalin fixative for around 15 min may be sufficient. On the other hand, if procedural time and resources are less of a constraint, then using a washout step and choosing a fixative solution optimized for the desired downstream studies may be helpful. If severe vascular pathology such as hemorrhage is present in a focal area of the brain, then perfuse the contralateral hemisphere or avoid perfusion fixation in that brain altogether. Overall, perfusion fixation is an appropriate method to use for higher quality fixation of deep structures and possible improved immunogenicity. The overall choice will depend upon the goals and available resources of each investigator and brain bank.

## Additional files

Additional file 1: PRISMA checklist for the study. (PDF 109 kb)

Additional file 2: Database search methods. (PDF 48 kb)

Additional file 3: Data extraction form and study appraisal tool. (PDF 50 kb)

## Data Availability

All data analyzed during this study are included in this published article and its supplementary information files. The code used to generate Fig. 2 is available at https://github.com/andymckenzie/HBPF_Review.

## Abbreviations

GRADE: Grading of Recommendations, Assessment, Development, and Evaluations
IJV: Internal jugular vein
JBI: Joanna Briggs Institute
kPa: Kilopascals
mOsm: Milliosmole
NR: Not reported
PBS: Phosphate-buffered saline
PCoA: Posterior communicating artery
PMI: Postmortem interval
PRISMA: Preferred Reporting Items for Systematic Reviews and Meta-Analyses
