# Supplementary material for: Perfusion fixation in brain banking: a systematic review
Source: Acta Neuropathol Commun. 2019 Sep 5;7:146. doi: 10.1186/s40478-019-0799-y (PMC6728946; doi:10.1186/s40478-019-0799-y)
Supplement: Supplementary file 1 — PRISMA checklist for the study. (PDF 109 kb) [file 40478_2019_799_MOESM1_ESM.pdf]

PRISMA 2009 Checklist

Section/topic	#	Checklist item	Page # and text excerpt
**TITLE**			
Title	1	Identify the report as a systematic review, meta-analysis, or both.	Page 1: "…a systematic review…"
**ABSTRACT**			
Structured summary	2	Provide a structured summary including, as applicable: background; objectives; data sources; study eligibility criteria, participants, and interventions; study appraisal and synthesis methods; results; limitations; conclusions and implications of key findings; systematic review registration number.	Page 2, section "Abstract"
**INTRODUCTION**			
Rationale	3	Describe the rationale for the review in the context of what is already known.	Pages 2-4, section "Introduction"
Objectives	4	Provide an explicit statement of questions being addressed with reference to participants, interventions, comparisons, outcomes, and study design (PICOS).	Page 4: "we aimed to identify studies that have performed perfusion fixation for human brain banking and performed a qualitative synthesis of their methodologies"
**METHODS**			
Protocol and registration	5	Indicate if a review protocol exists, if and where it can be accessed (e.g., Web address), and, if available, provide registration information including registration number.	Page 4: "The protocol for the review and updated versions of it the can be found at Open Science Framework (https://osf.io/cv3ys/)"
Eligibility criteria	6	Specify study characteristics (e.g., PICOS, length of follow-up) and report characteristics (e.g., years considered, language, publication status) used as criteria for eligibility, giving rationale.	Page 5, section "Eligibility criteria"
Information sources	7	Describe all information sources (e.g., databases with dates of coverage, contact with study authors to identify additional studies) in the search and date last searched.	Page 4, section "Search methods"
Search	8	Present full electronic search strategy for at least one database, including any limits used, such that it could be repeated.	Supplementary File 2 contains full electronic search for all databases used
Study selection	9	State the process for selecting studies (i.e., screening, eligibility, included in systematic review, and, if applicable, included in the meta-analysis).	Page 5, section "Study selection"
Data collection process	10	Describe method of data extraction from reports (e.g., piloted forms, independently, in duplicate) and any processes for	Page 5, section "Data collection"<br>Supplementary File 3 for the questionnaire used

		obtaining and confirming data from investigators.	
Data items	11	List and define all variables for which data were sought (e.g., PICOS, funding sources) and any assumptions and simplifications made.	Page 5, "The data variables that were extracted are…"<br>Also page 7, "One assumption made during the data extraction phase was that if…"
Risk of bias in individual studies	12	Describe methods used for assessing risk of bias of individual studies (including specification of whether this was done at the study or outcome level), and how this information is to be used in any data synthesis.	Page 6, "Study appraisal"<br>Supplementary File 3 for the study appraisal tool used
Summary measures	13	State the principal summary measures (e.g., risk ratio, difference in means).	Not applicable – only a narrative summary was performed
Synthesis of results	14	Describe the methods of handling data and combining results of studies, if done, including measures of consistency (e.g., I^2) for each meta-analysis.	Not applicable – only a narrative summary was performed

Section/topic	#	Checklist item	Page # and text excerpt
Risk of bias across studies	15	Specify any assessment of risk of bias that may affect the cumulative evidence (e.g., publication bias, selective reporting within studies).	Page 6, section "Qualitative data analysis", description of the GRADE method
Additional analyses	16	Describe methods of additional analyses (e.g., sensitivity or subgroup analyses, meta-regression), if done, indicating which were pre-specified.	Not applicable – only a narrative summary was performed
**RESULTS**			
Study selection	17	Give numbers of studies screened, assessed for eligibility, and included in the review, with reasons for exclusions at each stage, ideally with a flow diagram.	Pages 7-8, "We screened 4489 abstracts…" and Figure 1
Study characteristics	18	For each study, present characteristics for which data were extracted (e.g., study size, PICOS, follow-up period) and provide the citations.	Pages 9-10, Table 1, and other tables
Risk of bias within studies	19	Present data on risk of bias of each study and, if available, any outcome level assessment (see item 12).	Page 28, Figure 3, Table 8

Results of individual studies	20	For all outcomes considered (benefits or harms), present, for each study: (a) simple summary data for each intervention group (b) effect estimates and confidence intervals, ideally with a forest plot.	(a) Pages 26-27, Table 7<br>(b) – Not applicable, only a qualitative summary was performed
Synthesis of results	21	Present results of each meta-analysis done, including confidence intervals and measures of consistency.	Not applicable, only a qualitative summary was performed
Risk of bias across studies	22	Present results of any assessment of risk of bias across studies (see Item 15).	Page 29, paragraph "For the outcome of subjective histology quality immediately following the procedures, we assigned an evidence grade of moderate quality…"
Additional analysis	23	Give results of additional analyses, if done (e.g., sensitivity or subgroup analyses, meta-regression [see Item 16]).	Not applicable, only a qualitative summary was performed
**DISCUSSION**			
Summary of evidence	24	Summarize the main findings including the strength of evidence for each main outcome; consider their relevance to key groups (e.g., healthcare providers, users, and policy makers).	Pages 31-32, section "Conclusions"
Limitations	25	Discuss limitations at study and outcome level (e.g., risk of bias), and at review-level (e.g., incomplete retrieval of identified research, reporting bias).	Pages 30-31, "Limitations of this review"
Conclusions	26	Provide a general interpretation of the results in the context of other evidence, and implications for future research.	Pages 29-30, "Comparison to other reviews"<br>Page 31, "Recommendations for further research"
**FUNDING**			
Funding	27	Describe sources of funding for the systematic review and other support (e.g., supply of data); role of funders for the systematic review.	Page 32, "Funding"

From: Moher D, Liberati A, Tetzlaff J, Altman DG, The PRISMA Group (2009). Preferred Reporting Items for Systematic Reviews and Meta-Analyses: The PRISMA Statement. PLoS Med 6(7): e1000097. doi:10.1371/journal.pmed1000097

For more information, visit: **www.prisma-statement.org**.
