# Supplementary material for: Perfusion fixation in brain banking: a systematic review
Source: Acta Neuropathol Commun. 2019 Sep 5;7:146. doi: 10.1186/s40478-019-0799-y (PMC6728946; doi:10.1186/s40478-019-0799-y)
Supplement: Supplementary file 2 — Database search methods. (PDF 48 kb) [file 40478_2019_799_MOESM2_ESM.pdf]

Embase (Ovid)

Embase Classic+Embase 1947 to 2019 February 25
1556 results
1. exp central nervous system/
2. (brain* or cerebrum or encephalon).tw,kw.
3. (central adj4 nervous adj4 system*).tw,kw.
4. CNS.tw,kw.
5. or/1-4
6. organ perfusion/ or artery perfusion/ or brain perfusion/
7. perfusion/
8. perfusion*.tw,kw.
9. or/6-8
10. tissue fixation/
11. (fixation or preservation).tw,kw.
12. or/10-11
13. 5 and 9 and 12

Medline (Ovid)

Ovid MEDLINE(R) and Epub Ahead of Print, In-Process & Other Non-Indexed Citations, Daily and Versions(R) 1946 to February 25, 2019
1071 results

1. exp Central Nervous System/
2. (brain* or cerebrum or encephalon).tw,kw,kf.
3. (central adj4 nervous adj4 system*).tw,kw,kf.
4. CNS.tw,kw,kf.
5. or/1-4
6. exp Perfusion/
7. perfusion*.tw,kw,kf.
8. or/6-7
9. exp Tissue Preservation/
10. (fixation or preservation).tw,kw,kf.
11. or/9-10
12. 5 and 8 and 11

PubMed

1062 results as of 2/26/19
("Central Nervous System"[Mesh] OR (brain[tiab] OR brains[tiab] OR cerebrum[tiab] OR encephalon[tiab]) OR (central[tiab] AND nervous[tiab] system[tiab]) OR CNS[tiab]) AND ("Perfusion"[Mesh] OR (perfusion*[tiab])) AND ("Tissue Preservation"[Mesh] OR (fixation[tiab] OR preservation[tiab]))

Scopus

1543 results as of 2/25/19

Copy and paste:
(TITLE-ABS-KEY (brain* OR cerebrum OR encephalon) OR TITLE-ABS-KEY (central W/3 nervous W/3 system*) OR TITLE-ABS-KEY (cns)) AND (TITLE-ABS-KEY (perfusion*)) AND (TITLE-ABS-KEY (fixation OR preservation))
