# Supplementary material for: Perfusion fixation in brain banking: a systematic review
Source: Acta Neuropathol Commun. 2019 Sep 5;7:146. doi: 10.1186/s40478-019-0799-y (PMC6728946; doi:10.1186/s40478-019-0799-y)
Supplement: Supplementary file 3 — Data extraction form and study appraisal tool. (PDF 50 kb) [file 40478_2019_799_MOESM3_ESM.pdf]

Human Brain Perfusion Fixation Study Appraisal and Data Extraction Form

Methods Section

1. What is your name?

2. Study - first author last name/year

3. What was the country in which the study was performed?

4. What was the major purpose of the study? (E.g.: histology; forensic pathology; surgical training; gross anatomy; blood vessel anatomy).

5. What was the number of perfusion fixed brains?

6. What was the number of immersion fixed brains, if any? (Write "NA" if none.)

7. What were the donor exclusion criteria that would restrict the brains from being perfused fixed? (List any exclusion criteria mentioned as a separate bullet point.)

8. What was the overall tissue processing approach to the perfusion fixation?
In situ (brain in skull), neck dissection
In situ, head separated
Ex vivo (brain removed from skull), both hemispheres
Ex vivo, one hemisphere
Unclear/not reported
Other:

9. What was the cannula type used?

10. What were the other tissue processing steps performed prior to vascular access? (If necessary, list separate steps as separate bullet points.)

11. What were the vessels accessed for perfusion?

12. What was the method for driving perfusate flow?
Syringe
Pump
Gravity
Not recorded
Other:

13. What was the pre-fixative washout solution used? (If none, "NA")

14. What was the amount of pre-fixative washout solution perfused? (If none, "NA")

15. What was the time for the perfusion of the pre-fixative washout solution? (If none, "NA")

16. What was the fixative mixture?

17. What was the fixative buffer?

18. What was the amount of fluid perfused?

19. What was the time for fluid perfusion?

20. What was the perfusion pressure, if any was specified or recorded? (Or distance above the ground, if gravity perfusion was used.)

21. What were the tissue processing steps prior to post-fixation, if any?

22. What was the post-fixation procedure, if any?

23. What was the tissue processing procedure after post-fixation, if any?

24. What was the long-term storage procedure?

25. What were the metrics for fixation quality, if any?

26. What were the downstream assays or studies used or suggested, if any?

Study Appraisal

1. Does the study make a comparison between immersion and perfusion fixation?
Yes
No
Other:
If YES: Comparison Between Immersion and Perfusion Fixation On Brain Tissue Quality
The following questions relate to this comparison as it is made in the study.
If NO, skip these questions.

2. What were the metrics used for comparing perfusion vs immersion fixation?

3. What were the outcomes of the comparison between perfusion vs immersion fixation?

4. Were the participants included in any comparisons similar?
Yes
No
Unclear
Not applicable

5. Were the participants included in any comparisons receiving similar treatment, other than the exposure or intervention of interest?
Yes
No
Unclear
Not applicable

6. Was there a control group?
Yes
No
Unclear

Not applicable

7. Were there multiple measurements of the outcome both pre and post the intervention/exposure?
Yes
No
Unclear
Not applicable

8. Was follow up complete and if not, were differences between groups in terms of their follow up adequately described and analyzed?
Yes
No
Unclear
Not applicable

9. Were the outcomes of participants included in any comparisons measured in the same way?
Yes
No
Unclear
Not applicable

10. Were the outcome assessors blinded to the intervention used?
Yes
No
Unclear
Not applicable

11. Other than blinding, were the outcomes measured in a reliable way?
Yes
No
Unclear
Not applicable

12. Was appropriate statistical analysis used?
Yes
No
Unclear
Not applicable
